# 2-oxoglutarate-dependent dioxygenases and BAHD acyltransferases drive the structural diversification of orobanchol in Fabaceae plants

**DOI:** 10.3389/fpls.2024.1392212

**Published:** 2024-04-18

**Authors:** Masato Homma, Kiyono Uchida, Takatoshi Wakabayashi, Masaharu Mizutani, Hirosato Takikawa, Yukihiro Sugimoto

**Affiliations:** ^1^ Department of Agrobioscience, Graduate School of Agricultural Science, Kobe University, Kobe, Japan; ^2^ Department of Applied Biological Chemistry, Graduate School of Agricultural and Life Sciences, The University of Tokyo, Tokyo, Japan

**Keywords:** strigolactone, biosynthesis, orobanchol diversification, 2-oxoglutarate-dependent dioxygenase, BAHD acyltransferase

## Abstract

Strigolactones (SLs), a class of plant apocarotenoids, serve dual roles as rhizosphere-signaling molecules and plant hormones. Orobanchol, a major naturally occurring SL, along with its various derivatives, has been detected in the root exudates of plants of the Fabaceae family. Medicaol, fabacyl acetate, and orobanchyl acetate were identified in the root exudates of barrel medic (*Medicago truncatula*), pea (*Pisum sativum)*, and cowpea (*Vigna unguiculata*), respectively. Although the biosynthetic pathway leading to orobanchol production has been elucidated, the biosynthetic pathways of the orobanchol derivatives have not yet been fully elucidated. Here, we report the identification of 2-oxoglutarate-dependent dioxygenases (DOXs) and BAHD acyltransferases responsible for converting orobanchol to these derivatives in Fabaceae plants. First, the metabolic pathways downstream of orobanchol were analyzed using substrate feeding experiments. Prohexadione, an inhibitor of DOX inhibits the conversion of orobanchol to medicaol in barrel medic. The DOX inhibitor also reduced the formation of fabacyl acetate and fabacol, a precursor of fabacyl acetate, in pea. Subsequently, we utilized a dataset based on comparative transcriptome analysis to select a candidate gene encoding DOX for medicaol synthase in barrel medic. Recombinant proteins of the gene converted orobanchol to medicaol. The candidate genes encoding DOX and BAHD acyltransferase for fabacol synthase and fabacol acetyltransferase, respectively, were selected by co-expression analysis in pea. The recombinant proteins of the candidate genes converted orobanchol to fabacol and acetylated fabacol. Furthermore, fabacol acetyltransferase and its homolog in cowpea acetylated orobanchol. The kinetics and substrate specificity analyses revealed high affinity and strict recognition of the substrates of the identified enzymes. These findings shed light on the molecular mechanisms underlying the structural diversity of SLs.

## 1 Introduction

Strigolactones (SLs) are a type of plant apo-carotenoid that originates from all-*trans*-β-carotene. They serve as rhizosphere-signaling molecules and plant hormones (Aquino et al., 2021). These SLs, when exuded from plant roots, trigger the germination of seeds in root parasitic weeds belonging to the Orobanchaceae family, which includes the *Striga*, *Alectra*, *Orobanche*, and *Phelipanche* genera. This results in significant yield losses in key cereals, legumes, and vegetables across various regions of the world (Samejima and Sugimoto, 2018). Additionally, SLs induce hyphal branching in arbuscular mycorrhizal fungi, thereby fostering a beneficial symbiosis for plants (Akiyama et al., 2005). Internally, SLs act as regulators of plant architecture and development, functioning as a class of plant hormones (Gomez-Roldan et al., 2008; Umehara et al., 2008).

In terms of their chemical structure, SLs are categorized into canonical SLs, which consist of a tricyclic lactone (ABC ring) and a butenolide (D ring) connected with an enol ether bridge, and non-canonical SLs, where the ABC-ring system is incomplete (**Supplementary Figure S1A**). Canonical SLs are further subdivided into two types based on the configuration of the C-ring: strigol-type (**Supplementary Figure S1B**) with β-oriented C-rings, and orobanchol-type (**Supplementary Figure S1C**) with α-oriented C-rings. The structural variation is further increased by modifications of the A and B rings, such as hydroxylation, epoxidation, oxidation, and acetylation. To date, over 30 different SLs have been identified in the root exudates of various plants (Chesterfield et al., 2020).

Orobanchol, a major naturally occurring SL, has been detected in the root exudate of numerous plants, including monocots like rice (*Oryza sativa*) (Zhang et al., 2014) and dicots such as Sonalaceae (Wakabayashi et al., 2022), Fabaceae (Ueno et al., 2011), Cucurbitaceae (Khetkam et al., 2014), and Linaceae (Xie et al., 2009a). Regarding SL biosynthesis, enzyme reactions catalyzed by DWARF27 (D27), carotenoid cleavage dioxygenase 7 (CCD7), and CCD8 are known to convert all-*trans*-β-carotene to carlactone (Alder et al., 2012). The CYP711A subfamily is involved in the subsequent conversion of carlactone to carlactonoic acid (CLA) (Abe et al., 2014). Two distinct biosynthetic pathways of orobanchol from CLA have been elucidated. In rice, OsCYP711A2 and OsCYP711A3 convert CLA to 4-deoxyorobanchol (4DO) and then to orobanchol, respectively (Zhang et al., 2014). However, this biosynthetic pathway is presumed to be limited to certain monocot plants (Yoneyama et al., 2018). In dicot plants like tomato (*Solanum lycopersicum*) and cowpea (*Vigna unguiculat*a), an alternative pathway bypassing 4DO has been identified. In this pathway, CLA is converted to 18-oxo-CLA by CYP722C and subsequently to orobanchol by stereoselective BC-ring-forming factor (SRF) (Homma et al., 2023) (**Supplementary Figure S1D**).

Additionally, putative derivatives of orobanchol have been identified in the root exudates of various plants. For instance, downstream canonical SLs of orobanchol, namely 6,7-didehydroorobanchol and phelipanchols, have been identified in tomato (**Supplementary Figure S1C**) (Wakabayashi et al., 2022). These orobanchol derivatives were collectively termed didehydroorobanchol isomers (DDHs), with CYP712G1 identified as the enzyme responsible for the conversion of orobanchol to DDHs in tomato (Wang et al., 2022). The oxidation at the C7 position is suggested to be crucial for the conversion of orobanchol to DDHs in tomato (Wakabayashi et al., 2022). Furthermore, other plants also produce orobanchol derivatives biosynthesized through oxidation at the C7 position, such as 7-α/β-hydroxyorobanchyl acetate and 7-oxoorobanchyl acetate identified in cucumber (*Cucumis sativum*) (Khetkam et al., 2014) and flax (*Linum usitatissimum*) (Xie et al., 2009a), respectively (**Supplementary Figure S1C**).

**Figure 1:**
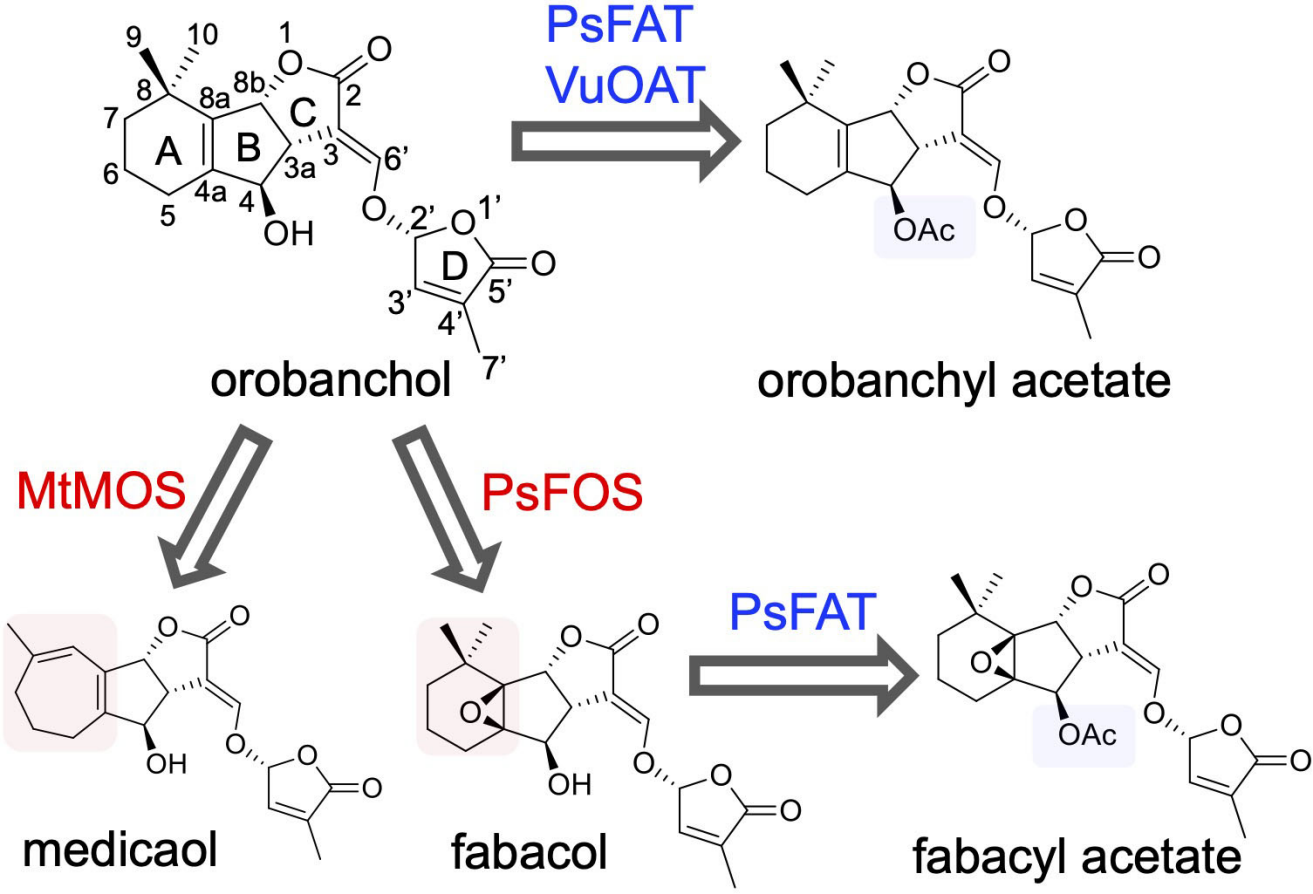
The diversification pathway of orobanchol in Fabaceae plants identified in this work (blue and red arrows). In barrel medic (*Medicago truncatula*), MtMOS converts orobanchol to medicaol. In pea (*Pisum sativum*), PsFOS converts orobanchol to fabacol. PsFAT acetylated fabacol and orobanchol, resulting in fabacyl acetate and orobanchyl acetate, respectively. In cowpea (*Vigna unguiculata*), VuOAT acetylated orobanchol.

Fabaceae plants, which encompass numerous important crops, exhibit several unique orobanchol derivatives. Medicaol, identified in the root exudates of barrel medic (*Medicago truncatula*), features a seven-membered A-ring instead of the typical six-membered A-ring found in SLs (Tokunaga et al., 2015). Similarly, fabacyl acetate, identified in the root exudates of pea (*Pisum sativum*), possesses an epoxide group at the A-ring and an acetoxy group at the C4 position (Xie et al., 2009b). Furthermore, orobanchyl acetate, characterized by an acetoxy group at the C4 position, was identified in the root exudates of cowpea (Ueno et al., 2011). These findings suggest distinct diversification pathways downstream of orobanchol in Fabaceae plants (**Figure 1**). However, the enzymes responsible for these diversifications are yet to be elucidated.

Despite advancements in SL research, the biological significance of the structural diversity of SLs remains unclear. Recent studies suggest that canonical SLs primarily function as signaling molecules in the rhizosphere (Ito et al., 2022), and investigations into their effects on the rhizosphere microbe community have been initiated (Schlemper et al., 2017; Kim et al., 2022). In a previous study, nodulation signaling pathway-1 (NSP1) and NSP2, which are GRAS-type transcription factors essential for rhizobium Nod factor-induced nodulation, are suggested to control not only downstream targets after rhizobium-induced signaling but also SL biosynthesis in barrel medic (Liu et al., 2011). Accordingly, molecular information on SL diversification in Fabaceae plants will accelerate studies on the rhizosphere microbiome.

The present study aimed to comprehensively elucidate the molecular and biochemical aspects of orobanchol diversification in Fabaceae plants. We conducted feeding experiments with potential biosynthetic precursors and inhibitors using plant hydroponics. Candidate genes of enzymes responsible for orobanchol derivatization were selected based on transcriptome data and co-expression analysis. Recombinant proteins of the candidate genes were heterologously expressed, and their enzyme activities were characterized. We report the identification of 2-oxoglutarate-dependent dioxygenases and BAHD acyltransferases responsible for the diversification of orobanchol to medicaol, fabacol, fabacyl acetate, and orobanchyl acetate. These findings will enable the manipulation of SL composition and shed light on the biological significance of SL diversification.

## 2 Materials and methods

### 2.1 General

The ^1^H NMR spectra were captured using a JNM-ECZ400S/L1 spectrometer (JEOL, Tokyo, Japan) in C_6_D_6_ at 400 MHz. The UV spectra were recorded on a V-630 spectrophotometer (JASCO, Tokyo, Japan). An LC-MS/MS system (Waters, Milford, MA, USA), which includes an Acquity UPLC H-Class and an Acquity TQD tandem mass spectrometer, was used to examine the SLs. Chromatographic separation was achieved using an ODS column (COSMOSIL 2.5C_18_-MS-II, 100 × 2.0 mm i.d., 2.5 µm) (Nacalai Tesque, Kyoto, Japan) maintained at a temperature of 30°C. The elution process was carried out in a linear gradient system of MeOH-H_2_O with 0.1% formic acid (50:50–100:0 in 20 min) at a flow rate of 0.2 ml/min. The TQD tandem mass spectrometer was operated under the following analytical conditions: in positive electrospray ionization mode, the capillary voltage was set to 3 kV, the source temperature was 120°C, the desolvation gas temperature was 350°C, the cone voltage was 30 V, and the collision energy was 20 eV. The nebulizer and desolvation N_2_ gas flow rates were set to 50 and 550 L/h, respectively. Data acquisition and analysis were conducted using MassLynx 4.2 software (Waters).

### 2.2 Chemicals

*Rac*-orobanchol was synthesized as described in a previous study (Uchida et al., 2023). *Rac*-2′-*epi*-orobanchol was procured from StrigoLab (Turin, Italy). The racemates were separated chiral-wise using HPLC on a CHIRALPAK IC (250 × 10 mm, 5 μm) (Daicel Corporation, Osaka, Japan), as reported by Nomura et al. (2013). Fabacol stereoisomers were synthesized by treating the corresponding orobanchol stereoisomer with mCPBA in CH_2_CL_2_, as outlined in a previous study (Xie et al., 2009b). This was followed by HPLC separation on a COSMOSIL 5C_18_-MS-II column (250 × 10 mm, 5 μm) (Nacalai Tesque) using MeOH-H_2_O with 0.1% formic acid (50:50) as the mobile phase at a flow rate of 5.0 ml/min. Subsequently, HPLC separation on a CHIRALPAK IC (250 × 10 mm, 5 μm) was used with EtOH containing 0.1% formic acid as the mobile phase at a flow rate of 0.8 ml/min. Orobanchyl acetate stereoisomers were synthesized by acetylating the corresponding orobanchol stereoisomer, following the method described previously (Ueno et al., 2011). Fabacyl acetate diastereomers were synthesized by epoxidizing the corresponding orobanchol acetate stereoisomer with mCPBA. Both orobanchyl acetate and fabacyl acetate stereoisomers were purified using HPLC, following the same procedures used for the purification of fabacol stereoisomers. The enzyme-reaction product of orobanchol by MtMOS was characterized as follows: A large-scale enzymatic reaction was performed using 50 ml of the reaction mixture with the same composition as described in the section of the *in vitro* enzyme assay. The enzyme reaction was conducted for 90 min using the crude enzyme prepared from 500 ml of *E. coli* culture medium and approximately 0.5 mg of *rac*-orobanchol as substrate. The reaction product was extracted with EtOAc (50 ml × 3), and the organic layer was dried over Na_2_SO_4_ and concentrated in vacuo. The large-scale reaction was conducted twice, and the reaction product was purified using HPLC, following the same procedure used for the purification of fabacol stereoisomers, yielding approximately 240 μg of the product. Its ^1^H-NMR spectrum was recorded in C_6_D_6_ (**Supplementary Figure S2**), which matched that reported for medicaol isolated from barrel medic (Tokunaga et al., 2015). 4DO was prepared as previously reported (Wakabayashi et al., 2023). The concentration of these chemicals was calculated using an absorption coefficient reported by Akiyama et al. (2010).

### 2.3 Plant material and hydroponics condition

Pea seeds (cv. *Akabana-Tsuruari*) and cowpea seeds (cv. *Blackeye*) were sourced from local suppliers, while barrel medic seeds (*Medicago truncatula* accession PI670016) were provided by the USDA. The pea and cowpea seeds were germinated on moistened filter paper in the dark for a period of 3 days. The barrel medic seeds were germinated on a 5 cm layer of vermiculite in a plastic strainer for a duration of 5 days. The seedlings of pea, cowpea, and barrel medic were then transferred to conical tubes and grown hydroponically in 50 mL of half-strength Hoagland nutrient solution at 23°C with a 16-h light/8-h dark photoperiod for 1 week, 2 weeks, and 4 weeks, respectively. For the feeding experiment, pea and barrel medic plants were grown in a half-strength Hoagland nutrient solution without phosphate. After 7 days and 11 days, respectively, the culture medium was replaced with fresh medium under phosphate-deficient conditions supplemented with 1 μM fluridone. After an additional 3 days, the culture medium was replaced again with fresh medium supplemented with 1 μM fluridone, the feeding substrate 40 nM *rac*-orobanchol or *rac*-orobanchyl acetate, and oxidation enzyme inhibitors 50 μM uniconazole-P or prohexadione dissolved in acetone. In the case of cowpea, plants grown in half-strength Hoagland were transferred to tap water with 1 μM fluridone. After 3 days, the hydroponic solution was replaced with fresh tap water supplemented with 1 μM fluridone and 40 nM *rac*-orobanchol. Twenty-four hours after feeding the substrate, each of the hydroponic solutions was subjected to Oasis HLB Vac Cartridge (Waters) to extract SLs as previously described (Moriyama et al., 2022), followed by LC-MS/MS analysis. For SL analysis in root exudates, plants were grown under the same conditions described above, but without fluridone and the oxidation enzyme inhibitors.

### 2.4 Gene expression analysis by reverse transcription-quantitative PCR (RT-qPCR)

cDNA templates were generated from RNAs isolated from the roots using the RNeasy Plant Mini Kit (Qiagen, Hilden, Germany) and ReverTra Ace qPCR RT Mix with genomic DNA Remover (TOYOBO, Osaka, Japan). The RNAs were extracted from the roots of three independent lines of hydroponically grown cowpea, barrel medic, and pea plants under both normal and phosphate-deficient conditions, as described in the previous section. These cDNA templates were then amplified using a StepOne (Applied Biosystems, Foster City, CA, USA) with PowerTrack SYBR Green Master Mix (Applied Biosystems). The primer sets, which are listed in **Supplementary Table S1**, were utilized to perform the qPCR analysis. The gene expression levels were normalized against the values of reference genes, and the relative gene expression levels were calculated based on the 2^−ΔΔCt^ method. The actin gene (ACT) (Wakabayashi et al., 2019), the ubiquitin gene (UBQ) (Liu et al., 2011), and the tubulin gene (TUB) (Guercio et al., 2022) served as reference genes for cowpea, barrel medic, and pea, respectively. The data acquisition and analysis were conducted using StepOne software v2.3 (Applied Biosystems).

### 2.5 Co-expression analysis by Confeito algorithm

Using the dataset from the Plant Bioinformatics Facility (https://urgi.versailles.inra.fr), genopea_counts_on_Genome_TPM.csv, which links the pea genome (Kreplak et al., 2019) to the pea transcriptome data (Alves-Carvalho et al., 2015), and the Confeito algorithm (Ogata et al., 2009) was used to perform gene co-expression analysis. This analysis revealed a local *PsSRF* (*Psat0ss8330g0240*) gene module; the genes within this module are detailed in **Supplementary Table S2**.

### 2.6 The expression of recombinant proteins in *E. coli*

The complete coding sequences (CDS) were sourced from the Phytozome and Ensemble Plants databases. The CDS of the candidate genes were amplified from root cDNA, which was prepared from barrel medic, pea, and cowpea under phosphate-deficient conditions. This amplification used primers with 15-bp overlap sequences for in-fusion cloning, as listed in **Supplementary Table S1**. The amplified DNA fragments were then inserted into the NdeI and KpnI restriction sites of the modified pCold III (Takara Bio, Shiga, Japan) expression vector using the In-Fusion HD cloning kit, as described by Wakabayashi et al. (2023). The constructed vectors were sequenced and introduced into the *E. coli* strain BL21 (DE3), which harbored the pGro12 plasmid (Nishihara et al., 1998), for the co-expression of the molecular chaperonins.

The transformed *E. coli* was cultured overnight at 37°C in LB medium supplemented with 100 μg/ml ampicillin and 50 μg/ml kanamycin. The overnight cultures were then diluted into LB medium containing 100 μg/ml ampicillin, 50 μg/ml kanamycin, and 0.5 mg/ml L-arabinose (for the induction of GroES/GroEL expression). The culture medium was incubated at 37°C until the OD600 reached 0.7. After chilling the cultures on ice for 30 min, protein expression was induced by adding 0.1 mM isopropyl β-D-thiogalactoside, and the cells were incubated for an additional 24 h at 15°C. The *E. coli* cells were collected by centrifugation and washed with phosphate-buffered saline. The crude enzymes were prepared as follows: the cells were disrupted by sonication in buffer A (50 mM potassium phosphate buffer (pH 7.4) and 20% (v/v) glycerol). After centrifugation at 10,000 g for 30 min at 4°C, the supernatant was used as the crude enzymes. A negative control was prepared from the cell harboring the empty vector.

### 2.7 Protein purification

*E. coli* cells, which expressed the recombinant proteins, were disrupted by sonication in a lysis buffer composed of 50 mM potassium phosphate buffer (pH 7.4), 500 mM KCl, 5 mM MgCl_2_, 0.1 mM EDTA, 0.1 mM DTT, 20% (v/v) glycerol, 1.0% (w/v) CHAPS, 5 mM ATP, and 25 mM imidazole. Following centrifugation at 10,000 g for 30 min at 4°C, the supernatant was applied to a TALON spin column (Takara Bio) that had been equilibrated with the lysis buffer. The proteins bound to the column were washed twice with 600 μl of the lysis buffer and then eluted twice with 200 μl of an elution buffer. This elution buffer was composed of 50 mM potassium phosphate buffer (pH 7.4), 500 mM KCl, 0.1 mM EDTA, 0.1 mM DTT, 20% (v/v) glycerol, 1.0% (w/v) CHAPS, and 500 mM imidazole. The eluent was then desalted using buffer A (50 mM potassium phosphate buffer (pH 7.4) and 20% (v/v) glycerol) with a 10 kDa cut-off Amicon Ultra concentrator (Merck Millipore, Burlington, MA, USA). Protein purification was confirmed by SDS-PAGE (**Supplementary Figure S3A**). The purified proteins were stored at −80°C. The protein concentration was estimated using the Pierce BCA Protein Assay Kit, following the manufacturer’s instructions (Thermo Fisher Scientific, Waltham, MA, USA).

### 2.8 *In vitro* enzyme assay

The DOX enzyme assay was conducted with a reaction mixture (50 μl) that included 50 mM potassium phosphate buffer (pH 7.4), 5 mM 2-oxoglutaric acid, 10 mM sodium ascorbate, 200 μM FeSO_4_, and either the crude enzymes or the purified enzymes. The BAHD acyltransferase enzyme assay was performed with a reaction mixture (50 μl) that consisted of 50 mM citrate buffer (pH 6.0), 200 μM acetyl-CoA, and either the crude enzymes or the purified enzymes. The identification assay for MtMOS, PsFOS, PsFAT, and VuOAT was conducted using the crude enzymes and 1 μM orobanchol or fabacol as substrates. For the kinetics analysis of the purified enzymes of MtMOS, PsFOS, and VuOAT, the concentration of orobanchol ranged from 0.05 to 1 μM. For the kinetics analysis of the PsFAT crude enzyme, the concentration of fabacol and orobanchol ranged from 0.25 to 10 μM. The enzymatic reaction was initiated by the addition of a substrate and conducted at 30°C for 10 min. The reaction products were extracted twice using 100 μl of EtOAc. The collected organic phase was evaporated, and the residues dissolved in acetonitrile were analyzed by LC-MS/MS. The standard curves for quantification were plotted using fabacol, fabacyl acetate, orobanchyl acetate, and medicaol standards as described in the section on chemicals. *K*_m_ and *K*_cat_ values were obtained using GraphPad Prism version 10 (**Supplementary Figure S3B**). For the analysis of substrate specificity of the purified enzymes (MtMOS, PsFOS, and VuOAT) and the crude enzyme of PsFAT, the enzymatic reaction using 0.2 μM substrates (orobanchol stereoisomers, fabacol stereoisomers, orobanchyl acetate, and 4DO) was conducted at 30°C for 20 min.

### 2.9 Phylogenetic tree analysis

The amino acid sequences utilized for the phylogenetic analysis were sourced from three databases: the National Center for Biotechnology Information, Phytozome, and Ensemble Plants. These sequences were aligned using the ClustalW tool. A phylogenetic tree was then constructed using the maximum-likelihood method, specifically employing the LG model with gamma-distributed rate variation among sites. Bootstrap statistics were computed using 1,000 replicates to ensure the robustness of the tree. All these phylogenetic analyses were conducted using the MEGA software, version 11 (Tamura et al., 2021).

## 3 Results

### 3.1 Metabolic pathway downstream of orobanchol in cowpea, barrel medic, and pea

**Figure 2:**
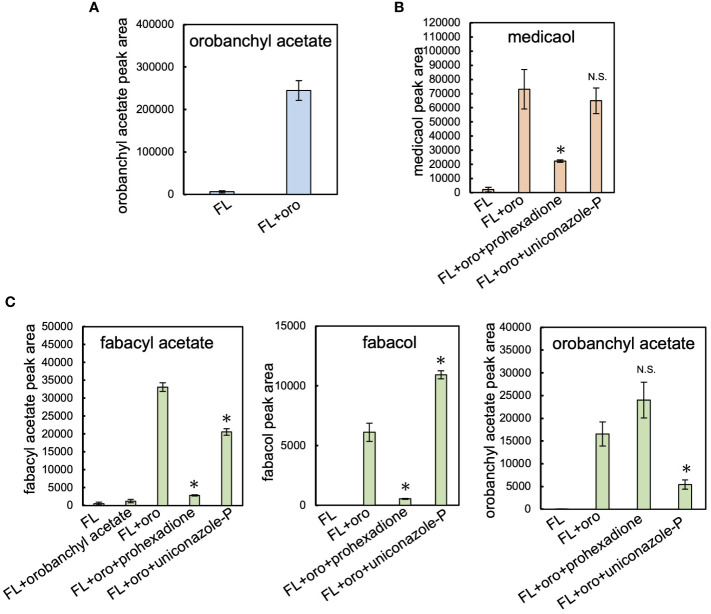
Feeding experiment in cowpea, barrel medic, and pea. **(A)** The level of orobanchyl acetate in cowpea root exudate supplemented with orobanchol. **(B)** The conversion of orobanchol to medicaol in barrel medic treated with oxidation enzyme inhibitors. Uniconazole-P and prohexadione are inhibitors of CYP and DOX, respectively. **(C)** The conversion of feeding substrates by pea treated with oxidation enzyme inhibitors to fabacyl acetate, fabacol, and orobanchyl acetate. Data are the means of three replicates ± SE. FL and oro denote fluridone and orobanchol, respectively. An asterisk indicates a significant difference (*p < 0.05, Student’s t test): N.S. means not significant between orobanchol feeding experiments with and without oxidation enzyme inhibitors. The peak area of each SL estimated by LC-MS/MS analysis was used for quantification.

Cowpea, barrel medic, and pea plants were cultivated hydroponically under phosphate-deficient conditions to stimulate the production of SLs (Yoneyama et al., 2007; López-Ráez et al., 2008). SLs extracted from root exudates were analyzed using LC-MS/MS. Orobanchyl acetate, medicaol, and fabacyl acetate were detected in the respective culture filtrates, along with orobanchol, as previously reported (**Supplementary Figure S4**) (Xie et al., 2009b; Ueno et al., 2011; Tokunaga et al., 2015). Each hydroponic system was treated with fluridone (Iseki et al., 2018), a phytoene desaturase inhibitor, which reduced SL accumulation to negligible levels. Feeding orobanchol to the hydroponics with fluridone revealed the conversion of the exogenously applied orobanchol to orobanchyl acetate, medicaol, and fabacyl acetate, respectively (**Figures 2A–C**).

In the pea root exudate, orobanchyl acetate was detected, but fabacol was not (**Supplementary Figure S4C**), as previously reported (Xie et al., 2009b). The exogenous application of orobanchyl acetate to the pea hydroponics did not significantly increase the accumulation of fabacyl acetate (**Figure 2C**). Notably, a significant amount of fabacol, which was below the detection limit in pea root exudate (**Supplementary Figure S4C**), accumulated in the hydroponics supplemented with orobanchol (**Figure 2C**; **Supplementary Figure S5B**). Therefore, the biosynthetic pathway from orobanchol to fabacyl acetate is presumed to proceed via fabacol (**Supplementary Figure S5A**).

The effects of uniconazole-P and prohexadione, inhibitors of cytochrome P450 (CYP) and 2-oxoglutarate and iron (II)-dependent dioxygenase (DOX) family enzymes, respectively, on the diversification of orobanchol were investigated. In barrel medic, the conversion of orobanchol to medicaol was significantly inhibited by prohexadione but not by uniconazole-P, suggesting the involvement of a DOX enzyme in the conversion (**Figure 2B**). In pea, the amount of fabacyl acetate and fabacol derived from orobanchol significantly decreased due to prohexadione (**Figure 2C**). The conversion of orobanchol to orobanchyl acetate and fabacyl acetate was inhibited by uniconazole-P (**Figure 2C**), with an increase in fabacol (**Figure 2C**). The uniconazole-P treatment seemed to inhibit the acetylation of fabacol and orobanchol by unknown mechanisms, although the inhibition of acyltransferase by uniconazole-P has not been reported. These results indicated that a DOX enzyme is responsible for the epoxidation of orobanchol in pea.

### 3.2 Identification of medicaol synthase in barrel medic

**Figure 3:**
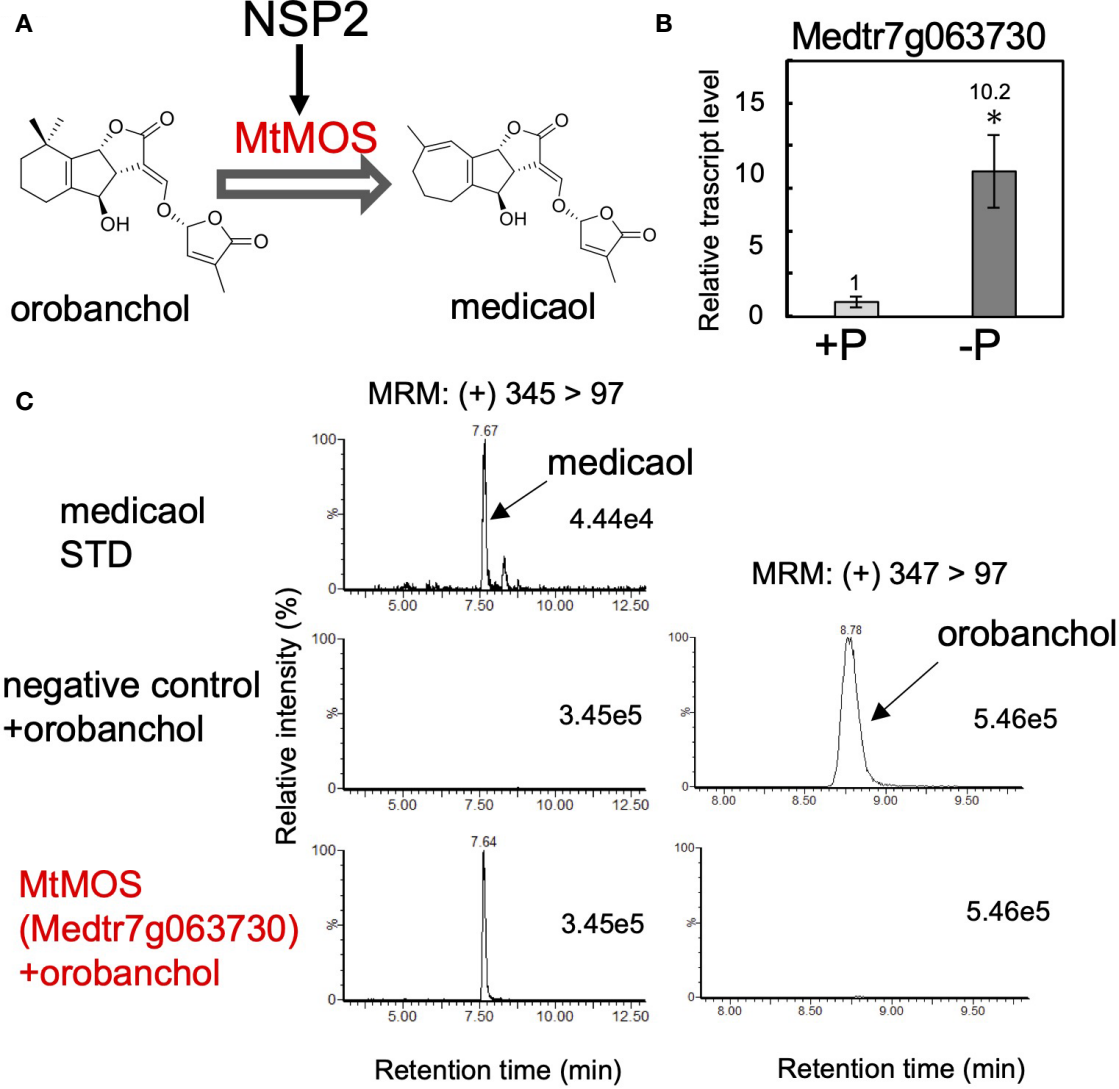
Identification of MtMOS. **(A)** The expression of MtMOS responsible for the conversion of orobanchol to medicaol is mediated by NSP2 in barrel medic. **(B)** The relative gene expression level of Medtr7g063730 under conditions with or without phosphate. Data are the means of three replicates ± SE. An asterisk indicates a significant difference (*p < 0.05, Student’s t test). **(C)** Enzyme activity of the recombinant protein of the candidate gene *Medtr7g063730* toward orobanchol. An enzyme assay was conducted using crude protein prepared from *E*. *coli* expressing candidate enzymes. The crude enzyme prepared from *E*. *coli* expressing a non-inserted empty vector was used as a negative control.

We expanded our research on orobanchol diversification to identify medicaol synthase (MOS), the DOX enzyme responsible for converting orobanchol to medicaol in barrel medic (**Figure 3A**). Previous studies on barrel medic mutants of NSP2, a GRAS-type transcription factor essential for rhizobium Nod factor-induced nodulation, reported a drastic decrease in the level of medicaol and an increase in orobanchol in the root exudate of the *nsp2* mutant (Liu et al., 2011; Tokunaga et al., 2015). These findings suggested that NSP2 regulates the expression of MOS, and impairment of NSP2 leads to the downregulation of *MOS* (**Figure 3A**). In a previous study, downregulated probes in *nsp2* roots compared to the wild type were listed based on microarray data. The top 100 downregulated probes were converted into gene IDs based on the barrel medic genome assembly (Mt4.0 v1), revealing that *Medtr7g063730* is the only gene encoding DOX (**Supplementary Table S3**). SL biosynthesis genes, including *MtD27* (*Medtr1g471050*), are known to be upregulated under phosphate-deficient conditions (Yoneyama et al., 2007; Liu et al., 2011). RT-qPCR analysis on the roots of barrel medic plants showed the upregulation of *Medtr7g063730* under phosphate deficiency (**Figure 3B**). These results suggest the involvement of this gene in SL biosynthesis as a MOS. We cloned *Medtr7g063730* from the cDNA of barrel medic roots, expressed the recombinant protein in *E. coli*, and performed enzyme assays using orobanchol as a substrate. The recombinant protein catalyzed the conversion of orobanchol to medicaol (**Figure 3C**), confirming that *Medtr7g063730* is the gene encoding MOS in barrel medic (**Figure 3A**).

DOX enzymes constitute the second-largest enzyme family in plants and are phylogenetically classified into DOXA, DOXB, and DOXC classes (Kawai et al., 2014). DOXC comprises 57 clades and is involved in the biosynthesis of plant secondary metabolites and plant hormones (Kawai et al., 2014). To understand the phylogeny of MtMOS among diverse DOX enzymes, a BLASTP search using the amino acid sequences of MtMOS as a query was conducted. Only Fabaceae plants, such as barrel medic, pea, soybean (*Glycine max*), red clover (*Trifolium pratense*), chickpea (*Cicer arietinum*), and birdsfoot trefoil (*Lotus japonicus*), have DOX enzymes homologous to MtMOS, sharing an amino acid sequence identity of more than 65%. DOX enzymes with the highest identities to MtMOS and its homologs are found in the DOXC54 clade, which contains lateral branching oxidoreductase (LBO) involved in SL biosynthesis (Kawai et al., 2014; Brewer et al., 2016). However, the amino acid identity is less than 45%. For instance, MtMOS shares amino acid identities of 43% and 44% with AtLBO (AT3G21420.1) from *Arabidopsis* (*Arabidopsis thaliana*) and its homolog from rice (LOC_Os01g70930.1), respectively. The phylogenetic tree shows that these Fabaceae DOX enzymes form a monophyletic clade, which is significantly different from the DOXC54 clade (**Supplementary Figure S6**). Thus, we designate these DOX enzymes represented by MtMOS as DOXC54-like 1.

### 3.3 Identification of fabacol synthase in pea

**Figure 4:**
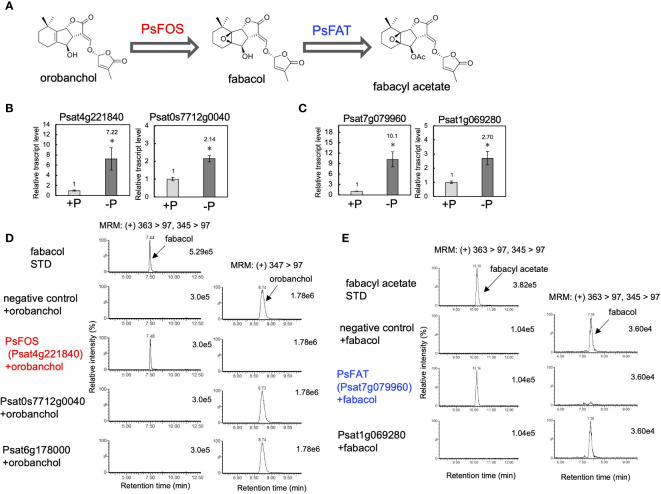
Identification of PsFOS and PsFAT. **(A)** The putative biosynthetic pathway of fabacyl acetate. PsFOS converts orobanchol to fabacol, which is acetylated by PsFAT. **(B)** The relative gene expression level of the candidate genes for PsFOS with or without phosphate. **(C)** The relative gene expression level of the candidate genes for PsFAT with or without phosphate. Data are the means of three replicates ± SE. An asterisk indicates a significant difference (*p < 0.05, Student’s t test). **(D)** Enzyme activity of the recombinant protein of the candidate gene for PsFOS. **(E)** Enzyme activity of the recombinant protein of the candidate gene for PsFAS. An enzyme assay was conducted using crude protein prepared from *E*. *coli* expressing candidate enzymes. The crude enzyme prepared from *E*. *coli* expressing a non-inserted empty vector was used as a negative control.

Following the results obtained from the feeding experiments conducted in pea plants, our research expanded to the identification of fabacol synthase (FOS), the DOX enzyme responsible for converting orobanchol to fabacol (**Figure 4A**). The biosynthetic pathway of orobanchol in pea has not yet been elucidated. However, homologs of VuCYP722C and VuSRF, which are responsible for the biosynthesis of orobanchol in cowpea (Homma et al., 2023), are found in the pea genome assembly (*Pisum sativum* Cameor genome v1a) (Kreplak et al., 2019). PsCYP722C (Psat4g095720) and PsSRF (Psat0ss8330g0240) exhibited high amino acid identities of 86% and 83%, respectively, with VuCYP722C and VuSRF, respectively. Thus, the same biosynthetic pathway of orobanchol as that of cowpea is suggested to operate in pea. Typically, biosynthetic genes of plant secondary metabolites exhibit similar expression patterns. Given that orobanchol serves as the substrate for PsFOS, we hypothesized that PsFOS would exhibit co-expression with PsSRF, responsible for orobanchol formation. The Plant Bioinformatics Facility (https://urgi.versailles.inra.fr) provided the dataset that links the pea genome (Kreplak et al., 2019) to the pea transcriptome, which was generated for a comprehensive gene expression atlas using 20 cDNA libraries prepared from various tissues collected at different developmental stages of pea plants grown under normal and low nitrate conditions (Alves-Carvalho et al., 2015). Nitrate deficiency was reported to induce SL biosynthesis in pea plants (Foo et al., 2013). Analysis of the dataset revealed exclusive expression of *PsSRF* (*Psat0ss8330g0240*) in roots (**Supplementary Figure S7**). Subsequently, co-expression analysis using the Confeito algorithm (Ogata et al., 2009) was conducted based on the dataset, resulting in the identification of the co-expression gene module of *PsSRF* (**Supplementary Table S2**). This gene module contained *PsD27* (*Psat6g058200*) and *PsCYP711A* (*Psat5g036360*), homologous genes to known SL biosynthesis genes, validating its efficacy in exploring candidate genes encoding PsFOS. Two DOX-encoding genes, *Psat4g221840* and *Psat0s7712g0040*, were included in the co-expression gene module (**Supplementary Table S2**, **Supplementary Figure S7**), with their expression upregulated by phosphate deficiency in roots (**Figure 4B**). Consequently, these two genes were selected as candidate genes encoding FOS. *Psat4g221840* and *Psat0s7712g0040* were cloned from cDNA synthesized from mRNA extracted from pea roots. Recombinant proteins of both genes were expressed in *E. coli*, and enzyme assays were conducted using orobanchol as the substrate. The recombinant protein of *Psat4g221840* facilitated the conversion of orobanchol to fabacol, while the other candidate gene did not yield any reaction product (**Figure 4D**), confirming *Psat4g221840* as the gene encoding FOS in pea (**Figure 4A**).

According to the pea genome, a homolog of PsFOS (Psat6g178000) shares a high amino acid identity of 83%. However, the recombinant protein of *Psat6g178000* did not exhibit enzyme activity toward orobanchol (**Figure 4D**). Other Fabaceae plants, including soybean, chickpea, barrel medic, red clover, and birdsfoot trefoil, possess homologs of PsFOS with amino acid identities exceeding 65%. DOX enzymes with the highest identities to PsFOS and its homologs are found in DOXC31, which encompasses functionally diverse enzymes identified from various plant species (Kawai et al., 2014), with amino acid identities reaching approximately 60%. The phylogenetic tree, including previously identified DOXC31 enzymes, indicated that these Fabaceae DOX enzymes are part of the DOXC31 clade (**Supplementary Figure S8**).

### 3.4 Identification of fabacol acetyltransferase in pea and orobanchol acetyltransferase in cowpea

The negligible conversion of orobanchyl acetate to fabacyl acetate and the successful conversion of orobanchol to fabacol (**Figure 2C**), combined with the identification of PsFOS in pea (**Figure 4D**), suggest that fabacyl acetate is biosynthesized from fabacol through the acetylation of the hydroxy group at the C4 position (**Figure 4A**). To identify fabacol acetyltransferase (FAT) in pea, we selected genes encoding BAHD acyltransferases, which constitute the enzyme family involved in the acylation of plant secondary metabolites, from the same gene module used for identifying PsFOS. This gene module contained two genes encoding BAHD acyltransferase (*Psat7g079960* and *Psat1g069280*) (**Supplementary Table S2**, **Supplementary Figure S7**), both of which were upregulated by phosphate deficiency in pea roots (**Figure 4C**). The candidate genes, *Psat7g079960* and *Psat1g069280*, were cloned from pea root cDNA. Recombinant proteins of the two genes were expressed in *E. coli*, and the enzyme assay was conducted with acetyl-CoA and fabacol as the acyl donor and acceptor, respectively. The recombinant protein of *Psat7g079960* catalyzed the conversion of fabacol to fabacyl acetate, while that of *Psat1g069280* did not (**Figure 4E**), indicating that *Psat7g079960* is the gene encoding FAT in pea (**Figure 4A**).

**Figure 5:**
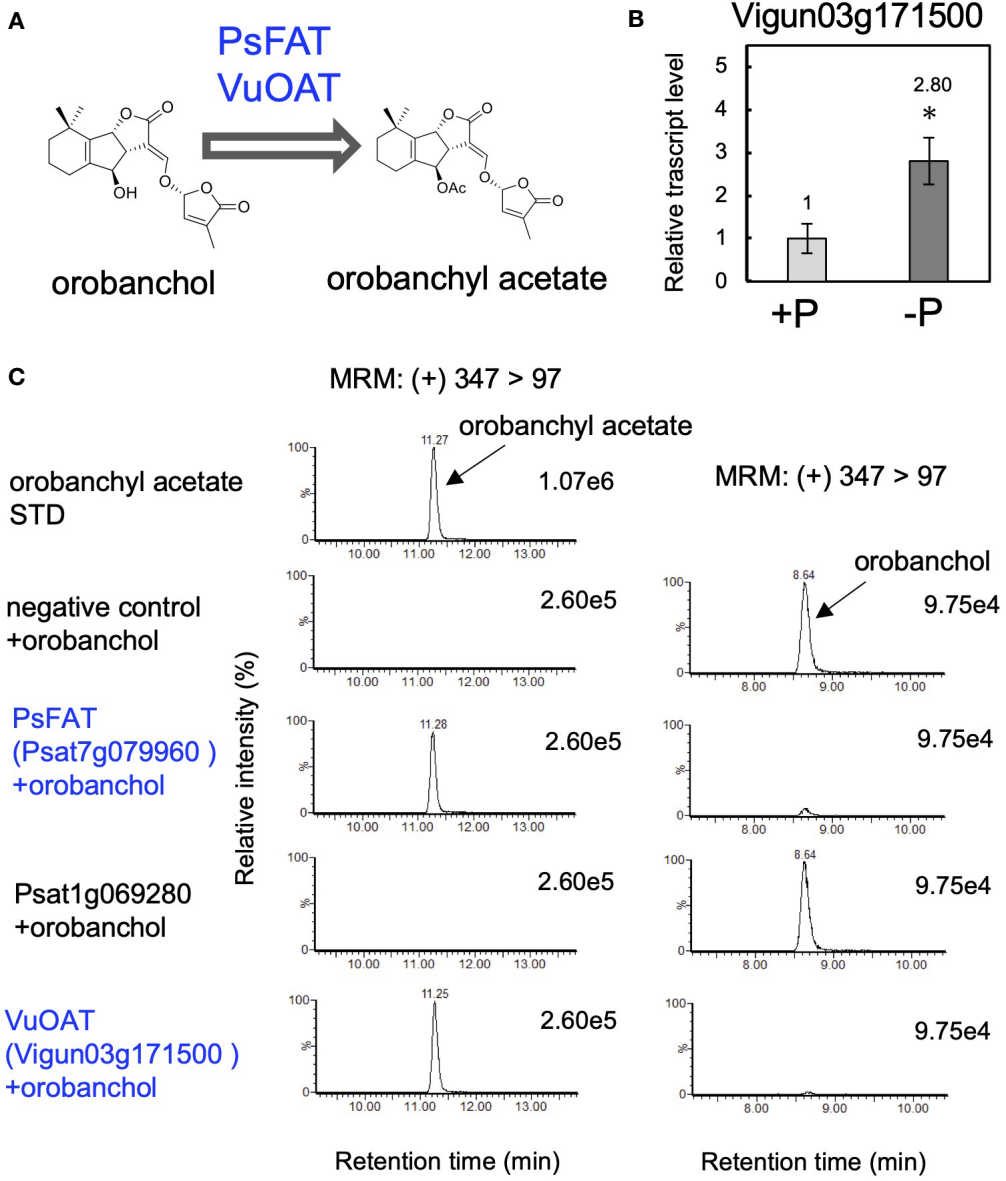
Identification of orobanchol acetyltransferases in pea and cowpea. **(A)** The acetylation of orobanchol by PsFAT and VuOAT was elucidated in this study. **(B)** The relative gene expression level of *Vigun03g171500* under conditions with or without phosphate. Data are the means of three replicates ± SE. An asterisk indicates a significant difference (*p < 0.05, Student’s t test). **(C)** Acetylation activity toward orobanchol of candidate enzymes. Enzyme assays were conducted using crude protein prepared from *E*. *coli* expressing PsFAT, Psat1g069280, and VuOAT, a homolog of PsFAT from cowpea. The crude enzyme prepared from *E*. *coli* expressing a non-inserted empty vector was used as a negative control.

In accordance with the detection of orobanchyl acetate in pea root exudates (**Supplementary Figure S4C**), PsFAT also acetylated orobanchol, while the recombinant protein of *Psat1g069280* did not (**Figure 5C**). This result suggests that the homolog of PsFAT in cowpea could acetylate orobanchol because both orobanchol and orobanchyl acetate are detected in cowpea root exudates (**Supplementary Figure S4A**). *Vigun03g171500*, the only gene encoding the homolog of PsFAT with 59% amino acid sequence identity, was upregulated by phosphate deficiency in cowpea roots (**Figure 5B**). The recombinant protein of this gene exhibited acetyltransferase activity toward orobanchol (**Figure 5C**), indicating that *Vigun03g171500* is the gene encoding orobanchol acetyltransferase (OAT) in pea (**Figure 5A**).

Previous studies have indicated that BAHD acyltransferases are phylogenetically divided into eight clades (Tuominen et al., 2011; Yuan et al., 2022). Phylogenetic analysis of PsFAT and VuOAT, the first identified acyltransferases involved in SL biosynthesis, revealed that only Fabaceae plants, including soybean, barrel medic, red clover, and birdsfoot trefoil, have BAHD acyltransferases sharing more than 60% amino acid identity with PsFAT or VuOAT. The phylogenetic tree, including previously identified BAHD acyltransferases from each clade, showed that PsFAT, VuOAT, and their homologs in Fabaceae plants belong to the IIIa clade of BAHD acyltransferases (**Supplementary Figure S9**), many of which are known to be involved in the acylation of alcohol acceptors using acetyl-CoA as the primary acyl donor (D’Auria, 2006; Yuan et al., 2022).

### 3.5 The biochemical characterization of MtMOS, PsFOS, PsFAT, and VuPAT

**Figure 6:**
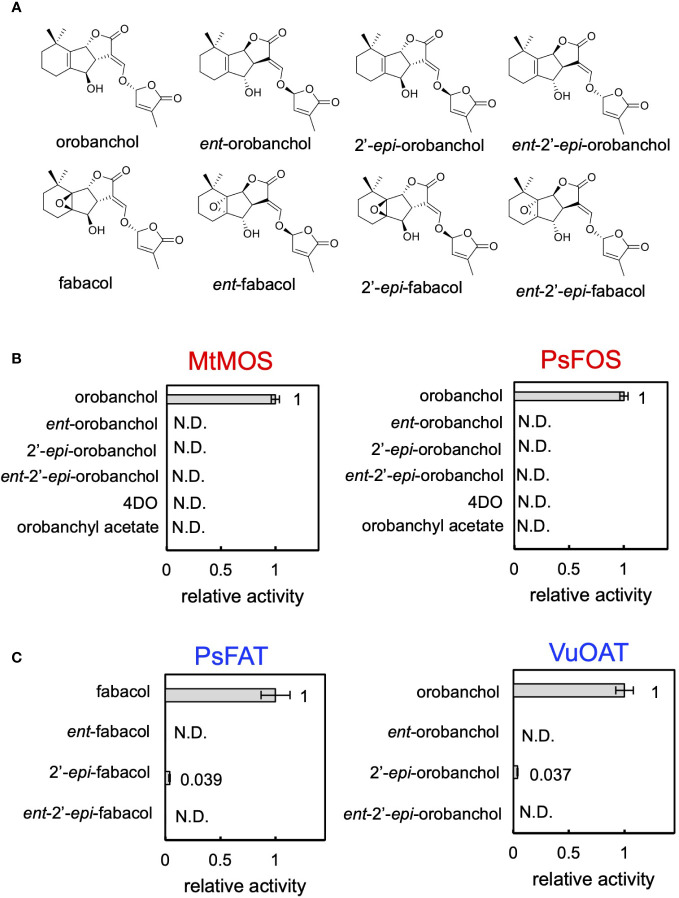
The substrate specificity of MtMOS, PsFOS, PsFAT, and VuOAT. **(A)** The chemical structure of the substrate used for the analysis, the stereoisomers of orobanchol and fabacol. **(B)** The enzyme activity of MtMOS and PsFOS toward the substrates, including orobanchol diastereomers, 4DO, and orobanchyl acetate. **(C)** The substrate specificity of PsFAT and VuOAT toward the diastereomers of fabacol and orobanchol, respectively. Relative enzyme activity of PsFAT toward the fabacol diastereomers normalized to the activity toward fabacol. The relative acetylation activity of VuOAT toward orobanchol diastereomers was normalized to the activity toward orobanchol. Data are the means of three replicates ± SE. N.D. means not detected.

Finally, we conducted biochemical characterization of the enzymes identified in this research. The recombinant proteins of DOX enzymes, MtMOS, and PsFOS were successfully purified (**Supplementary Figure S3A**), and their kinetic parameters for orobanchol were meticulously investigated. Notably, MtMOS and PsFOS displayed small apparent affinities (*K*_m_) for orobanchol, measuring at 0.070 and 0.162 μM, respectively (**Table 1A**). Due to the exceptionally high affinity of MtMOS toward orobanchol, its catalytic efficiency (*K*_cat_/*K*_m_) surpassed that of PsFOS, although their *K*_cat_ values were comparable (**Table 1A**). To explore substrate specificity, an enzyme assay was conducted using orobanchol stereoisomers (orobanchol, *ent*-orobanchol, 2′-*epi*-orobanchol, and *ent*-2′-*epi*-orobanchol) as substrates (**Figure 6A**). Remarkably, MtMOS and PsFOS exclusively exhibited enzyme activity toward orobanchol (**Figures 6B**, **Supplementary Figure S10A**). Furthermore, the conversion of 4DO and orobanchyl acetate by MtMOS and PsFOS was not observed (**Figures 6B**, **Supplementary Figures S10B, C**), indicating strict stereochemical recognition of orobanchol and the hydroxy group at the C4 position by these enzymes.

Table 1The kinetics analysis of the enzymes identified in this study.

**Table:** 

(A)				
enzyme	substrate	*K*_m_ (μM)	*K*_cat_ (/s)	*K*_cat_/*K*_m_ (/s·μM)
MtMOS	orobanchol	0.070 ± 0.013	2.66 × 10^−3^ ± 0.12 × 10^−3^	38.0 × 10^−3^ ± 7.2 × 10^−3^
PsFOS	orobanchol	0.162 ± 0.022	2.66 × 10^−3^ ± 0.12 × 10^−3^	16.4 × 10^−3^ ± 2.3 × 10^−3^
VuOAT	orobanchol	0.340 ± 0.094	51.4 × 10^−3^ ± 6 × 10^−3^	151 × 10^−3^ ± 45 × 10^−3^

**Table:** 

(B)			
enzyme	substrate	*K*_m_ (μM)	*V*_max_ (pM/s)
PsFAT	fabacol	0.287 ± 0.076	124.232 ± 6.500
PsFAT	orobanchol	2.277 ± 0.429	57.222 ± 3.588

(A) The kinetics parameters of three enzymes: MtMOS, PsFOS, and VuOAT. (B) The kinetics parameters of PsFOS. The data are the means of three replicates ± SE.

Moving on to the biochemical characterization of PsFAT, due to its low expression level in *E. coli*, crude proteins were utilized. In contrast, the recombinant VuOAT enzyme was successfully purified (**Supplementary Figure S3A**). The apparent affinities (*K*_m_) of PsFAT for fabacol and orobanchol were determined to be 0.287 and 2.277 μM, respectively (**Table 1B**). PsFAT exhibited a higher affinity for fabacol than for orobanchol, with the *V*_max_ value toward fabacol approximately double that toward orobanchol (**Table 1B**), indicating fabacol as the more preferred substrate for PsFAT. The *K*_m_ and *K*_cat_/*K*_m_ values of VuOAT for orobanchol were 0.340 μM and 151 × 10^−3^ (s^-1^·μM^-1^), respectively (**Table 1A**). The *K*_m_ value of VuOAT for orobanchol was comparable to that of PsFAT for fabacol and much smaller than that of PsFAT for orobanchol (**Tables 1A, B**).

To further probe substrate specificities, PsFAT and VuOAT were incubated with acetyl-CoA and four stereoisomers of fabacol and orobanchol, respectively (**Figure 6A**). Stereoisomers with β-oriented C-rings (*ent*-fabacol, *ent*-2′-*epi*-fabacol, *ent*-orobanchol, and *ent*-2′-*epi*-orobanchol) were not acetylated (**Figures 6C**, **Supplementary Figures S11A, B**). Weak acetylation activities toward 2′-*epi*-fabacol and 2′-*epi*-orobanchol were observed (**Supplementary Figures S11A, B**), approximately one twenty-fifth of the activity toward fabacol and orobanchol, respectively (**Figure 6C**). These results suggest that the C-ring configuration plays a crucial role in substrate recognition for these acyltransferases, while 2′-epimers are unlikely to occur in plants since naturally occurring SLs exclusively possess the *R* configuration at C2′ in the D ring.

## 4 Discussion

More than 30 SLs have been identified in the root exudates of various plants (Chesterfield et al., 2020), yet the evolutionary trajectory of enzymes involved in SL biosynthesis and its biological significance remain areas of ongoing investigation. The diverse oxidative modifications resulting from the evolution of CYP and DOX enzymes are major drivers of the structural diversity observed in secondary metabolites. Previous research has primarily focused on CYP family enzymes responsible for modifications on the A-ring of SLs, such as CYP728B35 (Wakabayashi et al., 2021), CYP706C37 (Li et al., 2023), CYP71AH (Wakabayashi et al., 2023), and CYP712G1 (Wang et al., 2022), in sorghum (*Sorghum bicolor*), maize (*Zea mays*), cotton (*Gossypium hirsutum*) and tomato, respectively. However, the identification of MtMOS and PsFOS in this work underscores the crucial role of DOX enzymes in the structural diversification of orobanchol within Fabaceae plants. To date, several DOX enzymes implicated in SL biosynthesis have been reported, although their precise functions remain uncertain. For example, LBO, classified into the DOXC54 clade, oxidizes methyl carlactonoate and is speculated to be involved in the biosynthesis of an unidentified SL that acts as a plant hormone inhibiting shoot branching (Brewer et al., 2016). Similarly, lotuslactone defective (LLD) (Mori et al., 2020) and Sb3500 (Yoda et al., 2023), classified into the DOXC55 clade, are involved in the biosynthesis of lotuslactone and 5-deoxystrigol in birdsfoot trefoil and sorghum, respectively. It is anticipated that further exploration of DOXC-class enzymes, particularly those phylogenetically related to the DOXC54 and DOXC55 clades, will lead to the identification of additional SL biosynthetic enzymes.

In the phylogenetic analysis, MtMOS is classified within the phylogenetic clade DOXC54-like 1, which is suggested to be phylogenetically linked to the DOXC54 clade (**Supplementary Figure S6**). While LBO, classified as DOXC54, remains highly conserved among seed plant species (Brewer et al., 2016), non-Fabaceae plants, such as Solanaceae, Cucurbitaceae, and Linaceae, known for producing orobanchol derivatives (Xie et al., 2009a; Kohlen et al., 2013; Wang and Bouwmeester, 2018), lack DOX enzymes homologous to DOXC54-like 1. DOX enzymes in these plants share only about 40% amino acid identity with DOXC54-like 1 enzymes. Consequently, DOXC54-like 1 enzymes are presumed to have evolved from DOXC54 clade enzymes in Fabaceae plants and are likely implicated in SL diversification specific to this plant family. Several Fabaceae plants reportedly produce putative DDHs, including medicaol (Yoneyama et al., 2008; Tokunaga et al., 2015; Galili et al., 2021). DOXC54-like 1 enzymes may have a role in the conversion of orobanchol to DDHs in this plant family. Furthermore, birdsfoot trefoil produces lotuslactone (Xie et al., 2019), a non-canonical SL sharing the same seven-membered A-ring as medicaol (**Supplementary Figure S1A**). In birdsfoot trefoil, a DOXC54-like 1 enzyme is probably involved in the A-ring expansion reaction of an unidentified substrate leading to lotuslactone. Therefore, further investigation into DOXC54-like 1 can shed more light on SL diversification in Fabaceae plants.

On the contrary, PsFOS is categorized under DOXC31, phylogenetically distinct from the adjacent clades of DOXC54 and DOXC55 (Kawai et al., 2014). The DOXC31 clade is one of the largest DOXC clades, encompassing functionally diverse enzymes responsible for lineage-specific metabolites (Kawai et al., 2014). For example, ZmBX6 (Jonczyk et al., 2008), CrD4H (Vazquez-Flota et al., 1997), AtGSLOH (Hansen et al., 2008), and E8/Sl27DOX (Akiyama et al., 2021), from maize, rosy periwinkle (*Catharanthus roseus*), *Arabidopsis*, and tomato, respectively, catalyze the hydroxylation of DIBOA glucoside, desacetoxyvindoline, 3-butenyl glucosinolate, and lycoperoside C. Additionally, SmTIIAS (Song et al., 2021) from redroot sage (*Salvia miltiorrhiza*), catalyzing the dehydrogenation of dihydrofuran to furan, is also included. Fabacyl acetate has solely been identified in pea, whereas DDHs including medicaol exhibit a broader distribution within Fabaceae plants (Yoneyama et al., 2008; Tokunaga et al., 2015; Galili et al., 2021). Thus, the unique diversification pathway catalyzed by PsFOS is suggested to have emerged through the evolution of DOXC31 in different lineages of DOXC55 and DOXC54, which are presumed to be involved in SL biosynthesis.

In Solanaceae and Fabaceae plants, orobanchol-type SLs are produced, but the rearrangement of the A-ring proceeds differently using different oxidation enzymes. As previously described, SlCYP712G1 has been identified as the enzyme responsible for converting orobanchol to DDHs in tomato. The DDHs catalyzed by SlCYP712G1 likely include 6,7-didehydroorobanchol, phelipanchol, and epiphelipanchol, as indicated by their chromatographic behavior compared to authentic SLs we identified (Wakabayashi et al., 2022; Wang et al., 2022). The elimination of the hydroxy group at the C7 position of 7-hydroxyorobanchol is presumed to be the key reaction, supported by the detection of putative 7-hydroxyorobanchol as a reaction product of SlCYP712G1 (Wakabayashi et al., 2022; Wang et al., 2022). Similarly, detailed analysis of a reaction product by MtMOS indicated a trace amount of putative monohydroxyorobanchol (**Supplementary Figure S12A**). The product could be an intermediate hydroxylated at the C9 or C10 position of orobanchol in the following reaction mechanism. A primary carbocation formed by the elimination of the hydroxy group could yield medicaol with the seven-membered A-ring through Wagner-Meerwein rearrangement followed by deprotonation, as previously proposed (Tokunaga et al., 2015) (**Supplementary Figure S12B**). However, recent studies have indicated radical-based ring expansion in the biosynthesis of tropolone (Doyon et al., 2020) and brevione (Yan and Matsuda, 2022), both catalyzed by DOX enzymes. Radical formation at the C9 or C10 position of orobanchol catalyzed by MtMOS may initiate the A-ring expansion (**Supplementary Figure S12C**). Based on this mechanism, the putative monohydroxyorobanchol detected in the enzyme reaction (**Supplementary Figure S12A**) may be a byproduct resulting from the rebound hydroxylation at the C9 or C10 position (**Supplementary Figure S12C**). Further studies are needed to gain more insight into this intriguing ring expansion mechanism.

In pea plants, orobanchol is presumed to be metabolized by two pathways: one involves the conversion to fabacyl acetate via fabacol, and the other involves the conversion to orobanchyl acetate (**Supplementary Figure S5A**). Considering the high affinity of PsFOS for orobanchol (**Figure 6B**, **Table 1A**) and the substrate preference of PsFAT for fabacol over orobanchol (**Table 1B**), the conversion of orobanchol to fabacyl acetate via fabacol is presumed to be the primary pathway of orobanchol derivatization in pea. This is consistent with the composition of SLs in pea root exudates supplemented with orobanchol, where the level of fabacyl acetate was much higher than orobanchyl acetate (**Supplementary Figure S5C**). The accumulation of a significant amount of fabacol in hydroponics supplemented with orobanchol (**Supplementary Figure S5B**) may be due to the exceedance of PsFAT’s conversion capacity toward an excess amount of fabacol formed by PsFOS from exogenously applied orobanchol. It is noteworthy that fabacol was identified for the first time in this work, whereas its occurrence was expected when fabacyl acetate was identified (Xie et al., 2009b), demonstrating the importance of substrate feeding experiments not only in elucidating the biosynthetic pathway but also in identifying biosynthetic precursors that are below the detection limit under normal cultural conditions.

Orobanchyl acetate has been identified in numerous Fabaceae plants (Yoneyama et al., 2008). Additionally, other SLs possessing an acetoxy group at the C4 position, such as fabacyl acetate and lotuslactone, have been isolated in pea and birdsfoot trefoil, respectively. Indeed, homologs of PsFAT and VuOAT are widely conserved in Fabaceae plants, and they can be potentially involved in the acetylation of the hydroxy group at the C4 position of SLs in Fabaceae plants. Alternatively, orobanchol-type SLs with an acetoxy group at the C4 position have been detected in plants outside the Fabaceae family. In Solanaceae plants, orobancyl acetate was detected in tomato and tobacco (*Nicotiana tabacum*) (Kohlen et al., 2013; Wang and Bouwmeester, 2018). Furthermore, 7-α/β-hydroxyorobanchyl acetate and 7-oxoorobanchyl acetate were isolated in cucumber (Khetkam et al., 2014) and flax (Xie et al., 2009a), respectively. However, tomato, tobacco, cucumber, and flax do not have homologs of PsFAT and VuOAT; BAHD acyltransferases in these plants share only ≤40% amino acid identity. Further studies are needed to identify the enzymes responsible for acetylation of the hydroxy group at the C4 position of orobanchol and its derivatives in plant species other than Fabaceae.

In conclusion, this study provides a comprehensive view of the biosynthesis pathway of canonical SLs downstream of orobanchol in Fabaceae plants (**Figure 1**) and offers a molecular basis to understand the structural diversity of SLs. Canonical SLs are suggested to primarily function in the rhizosphere as signaling molecules (Ito et al., 2022), and the structural diversification of orobanchol may play roles in plant-plant and plant-microbe interactions in the rhizosphere. The diversified orobanchol-type SLs may modulate the rhizosphere microbiome by selectively enriching specific bacteria beneficial to the secreting plants (Schlemper et al., 2017; Kim et al., 2022). The identification of genes responsible for orobanchol derivatization will enable genetic modification of SL composition in plants belonging to not only Fabaceae but also other plant families due to the wide distribution of orobanchol in plants. Studies on the rhizosphere microorganism profile using such plants with unique SL spectra will provide insight into the physiological significance of each of the diversified SLs. Results obtained from such studies can lead to agricultural applications for optimizing the rhizosphere environment.

## Data availability statement

The data in the study are included in the main article and supplementary material. The accession numbers for CDS of MtMOS, PsFOS, PsFAT, and VuOAT are LC800624, LC800625, LC800626, and LC800627, respectively.

## Author contributions

MH: Conceptualization, Data curation, Formal analysis, Investigation, Methodology, Software, Validation, Visualization, Writing – original draft, Writing – review & editing. KU: Formal Analysis, Investigation, Validation, Writing – review & editing. TW: Formal analysis, Writing – review & editing. MM: Writing – review & editing. HT: Project administration, Supervision, Writing – review & editing. YS: Conceptualization, Formal analysis, Funding acquisition, Project administration, Resources, Supervision, Writing – original draft, Writing – review & editing.
