# Supplementary material for: 2-oxoglutarate-dependent dioxygenases and BAHD acyltransferases drive the structural diversification of orobanchol in Fabaceae plants
Source: Front Plant Sci. 2024 Apr 18;15:1392212. doi: 10.3389/fpls.2024.1392212 (PMC11063326; doi:10.3389/fpls.2024.1392212)
Supplement: Supplementary file 1 [file DataSheet_1.pdf]

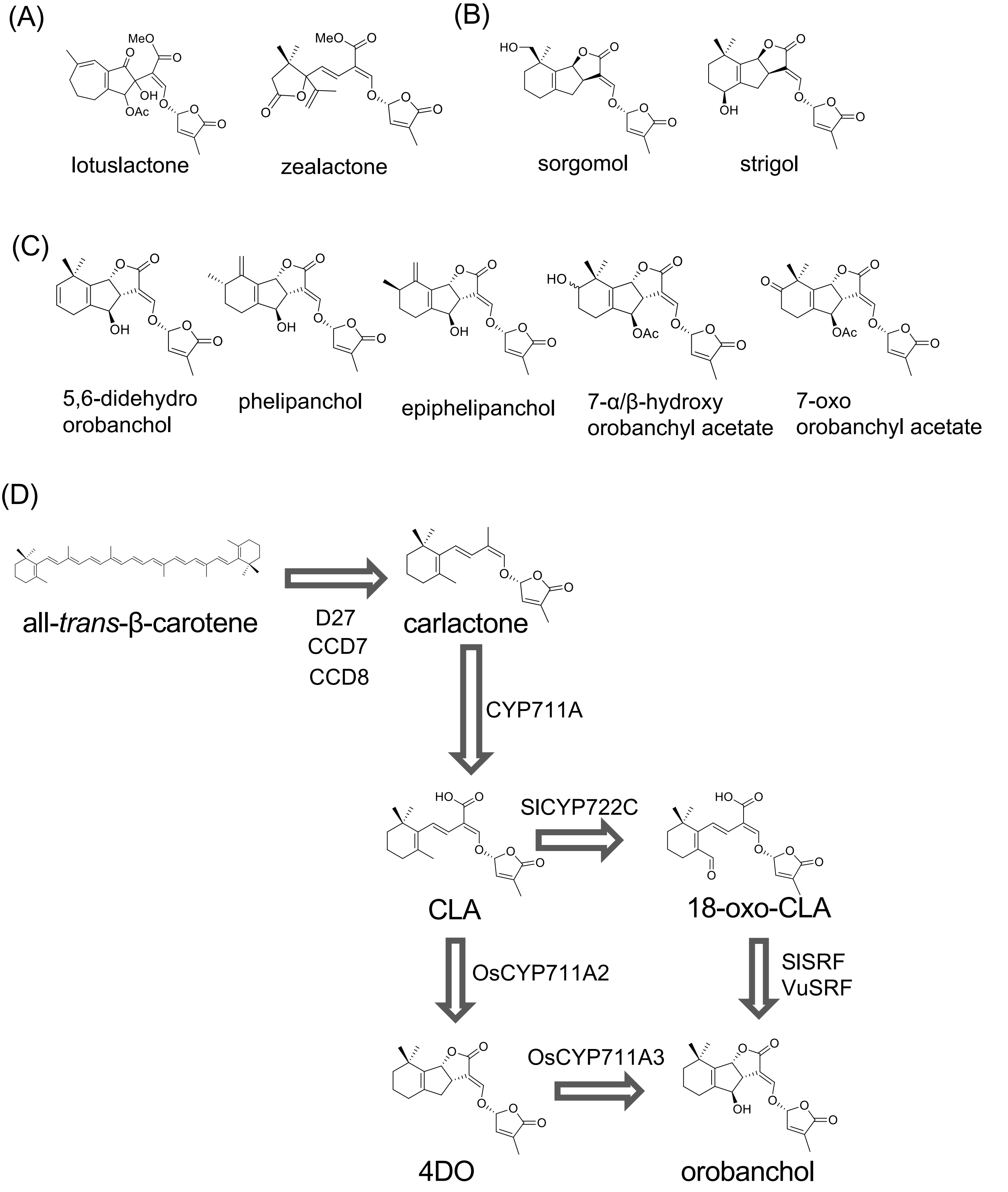

Figure S1. Chemical structures of naturally occurring SLs and previously established SL biosynthetic pathways: (A) Non-canonical SLs, (B) Strigol-type SLs with β-oriented C-ring, (C) Orobanchol-type SLs with α-oriented C-ring, (D) Biosynthetic pathways from all-*trans*-β-carotene to orobanchol.

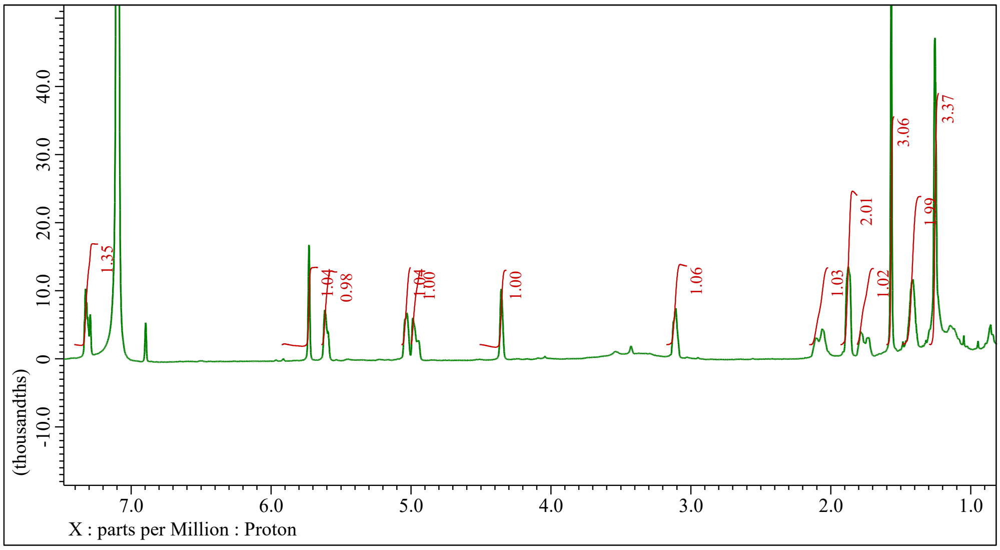

Figure S2. ^{1}H NMR spectrum of the enzyme product of MtMOS. The spectrum was recorded at 400 MHz in C_6D_6. Notably, this spectrum is identical to that of medicaol, as previously reported by Tokunaga et al. (2015).

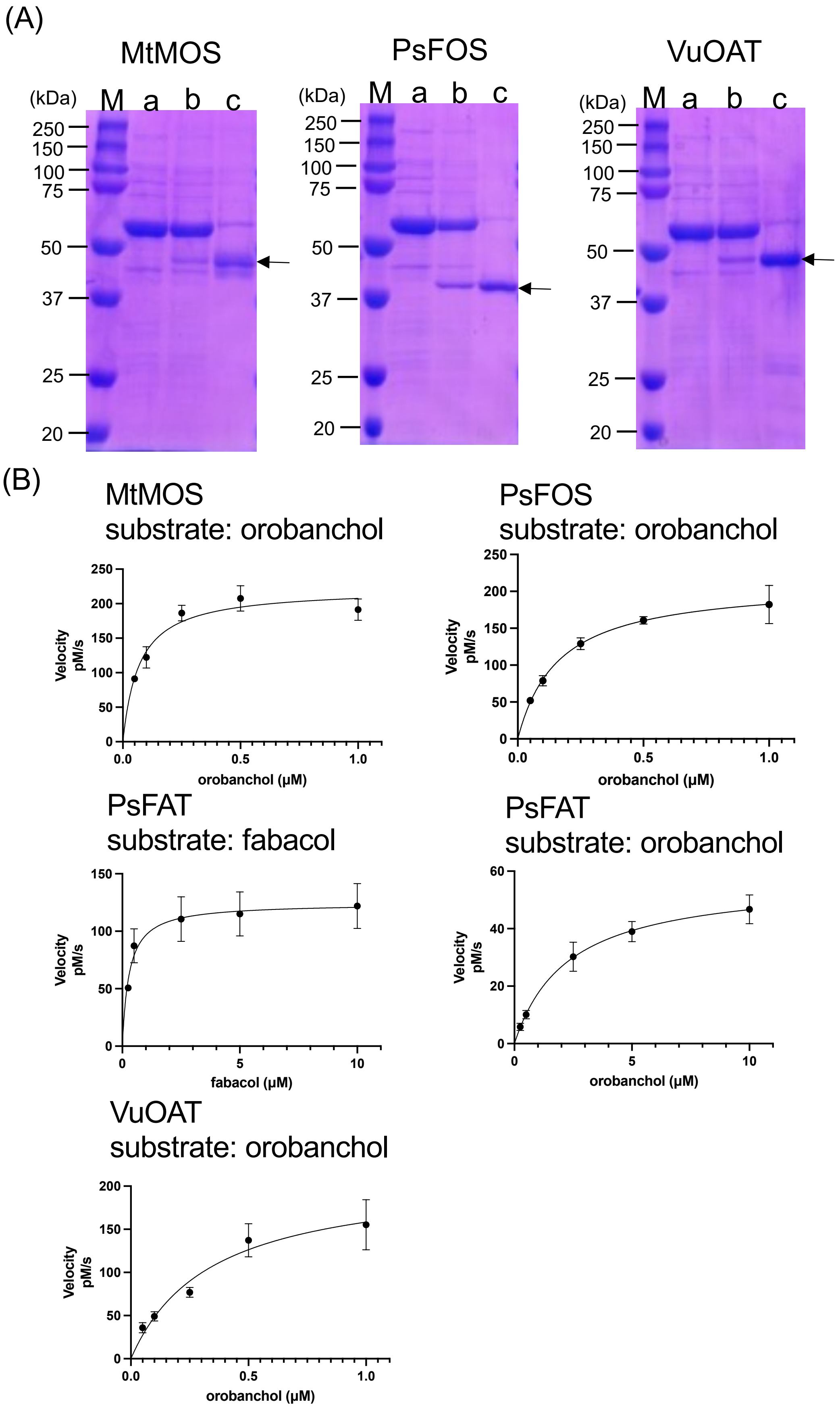

Figure S3. Biochemical analysis of MtMOS, PsFOS, PsFAT, and VuOAT. (A) Protein purification of the enzymes. The letters M, a, b, and c indicate the protein marker, the empty vector control, the crude protein before purification, and the purified protein, respectively. Arrow symbols indicate purified proteins. (B) Michaelis–Menten kinetics of MtMOS, PsFOS, PsFAT, and VuOAT. Data are the means of three replicates ± SE.

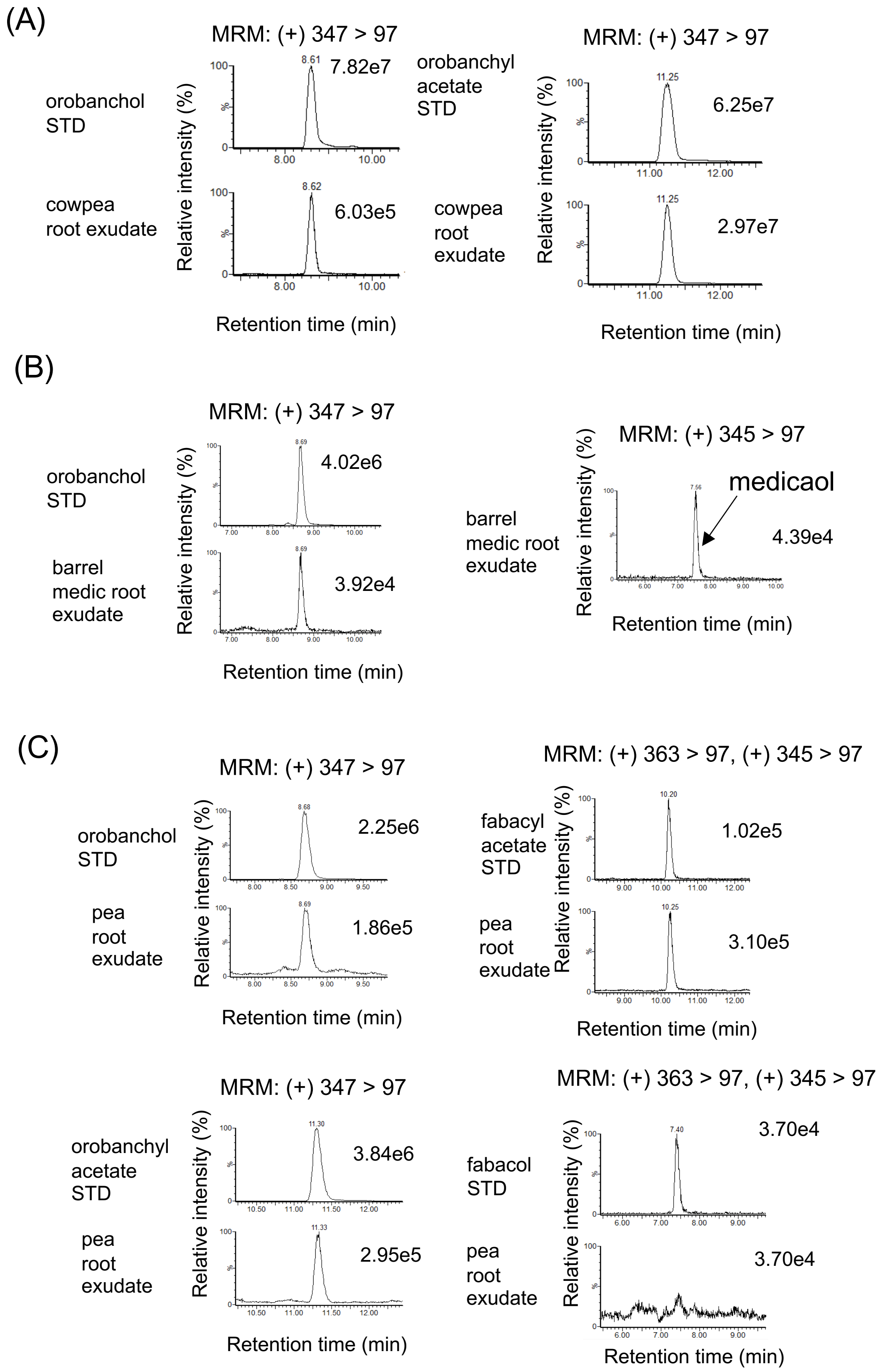

Figure S4. Analysis of root exudates in cowpea, barrel medic, and pea. (A) In cowpea root exudate, the detection of orobanchol and orobanchyl acetate is shown. (B) In barrel medic root exudate, the detection of orobanchol and medicaol is shown. Because no standard sample of medicaol was available, the enzyme-reaction product was identified as medicaol by NMR analysis and was identical to the compound detected in the root exudate. (C) In pea root exudate, the detection of orobanchol, orobanchyl acetate, and fabacyl acetate. The level of fabacol was below the detection limit.

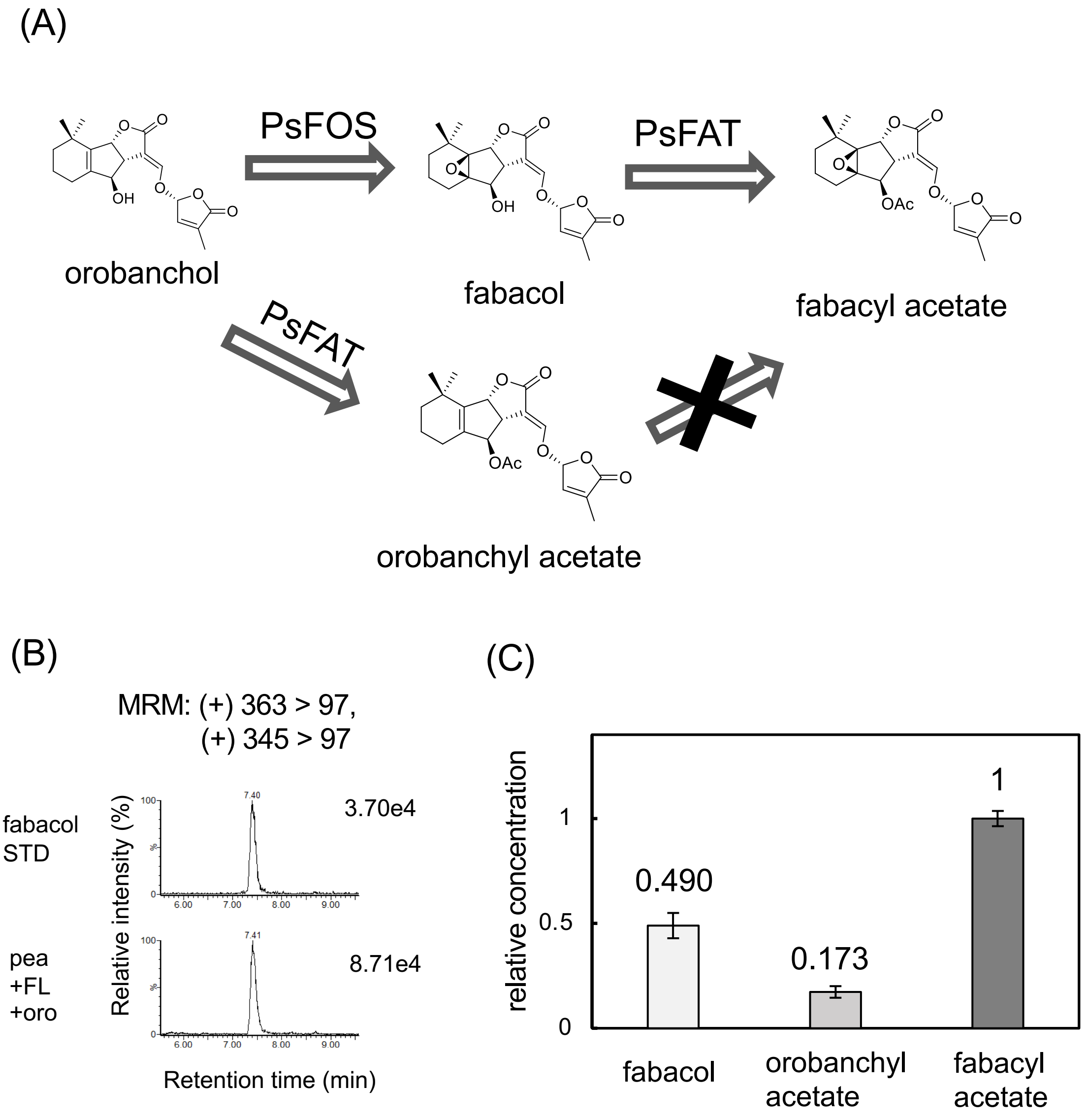

Figure S5. Detailed analysis of SLs in pea root exudates. (A) The SL biosynthetic pathway in pea proposed by this study. (B) The detection of fabacol in pea root exudate supplemented with orobanchol. (C) Quantitative comparision of fabacol, orobanchyl acetate, and fabacyl acetate in root exudate of pea treated with flulidone and supplemented with orobanchol. The data are the means of three replicates ± SE.

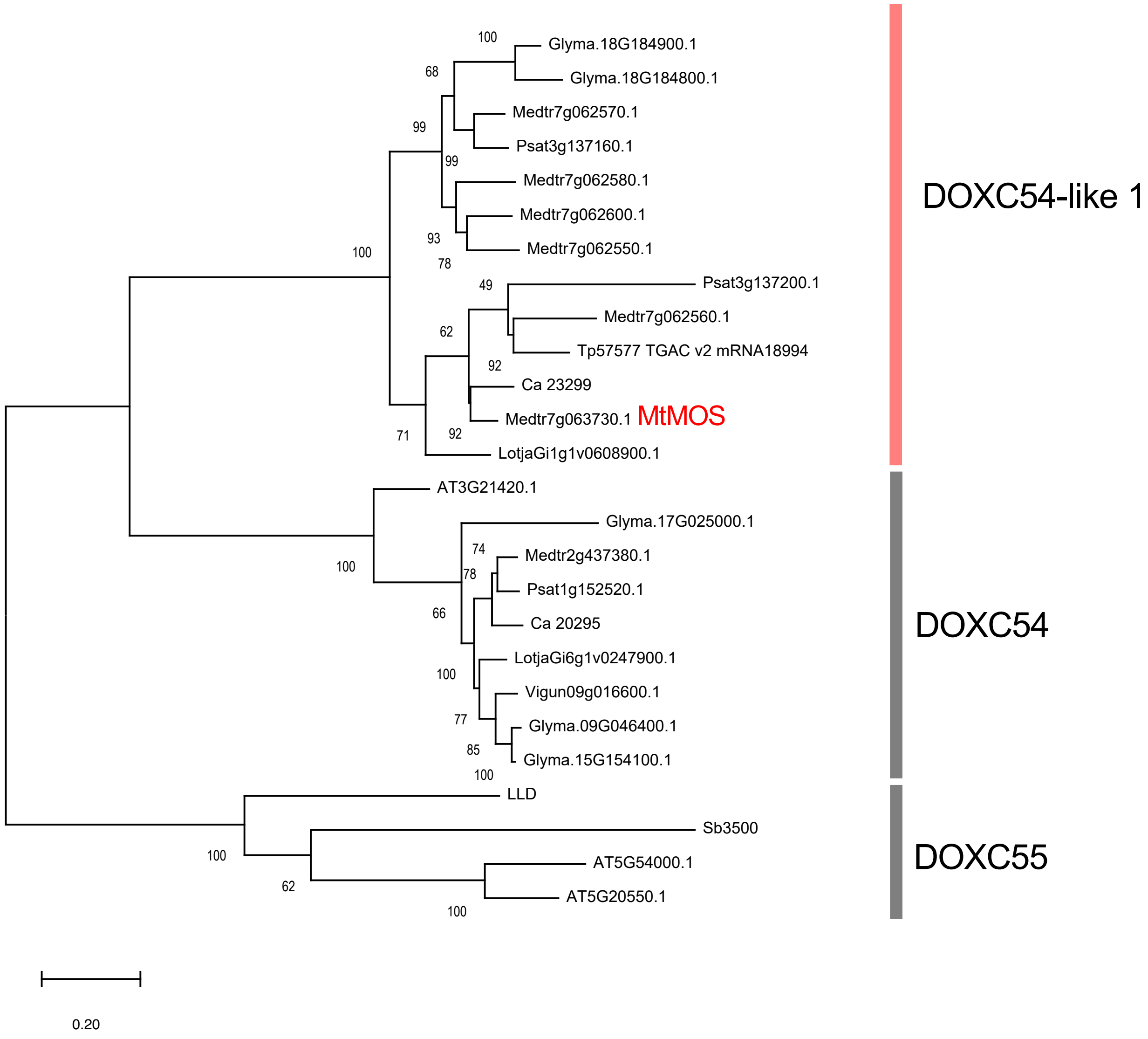

Figure S6. Phylogenetic analysis of MtMOS. Using the amino acid sequences of DOX enzymes showing amino acid identity greater than 65% to MtMOS, a phylogenic tree was constructed by the maximum-likelihood method. DOXC55 and DOXC54 enzymes were used as outgroups. Bootstrap values based on 1,000 replicates are shown at the branching points. The scale bar indicates 0.2 substitutions per position in the sequence.

gene ID	Psat0ss8330g0240	Psat4g221840	Psat0s7712g0040	Psat7g079960	Psat1g069280
anotation	SRF	DOX	DOX	BAHD acyltransferase	BAHD acyltransferase
co-expression efficiency	1	0.9849	0.9718	0.9849	0.9718
seeds_5dai	1.304738	4.355659	3.051195	0.205511	0.375628
Peduncle_C_LN	0.289297	0.481211	0.078189	0.154581	0.269737
Stem_BC_LN	0.60377	0.551056	1.089634	0.45021	0.177213
Tendril_BC_LN	0.421616	0.558693	0.071373	0.040463	0.823928
Shoot_A_HN	0.136186	0.235155	0	0.018875	0.099475
RootSys_A_HN	12.228299	13.19749	66.140107	8.671859	4.210777
Shoot_A_LN	0.135153	0.358269	0.107035	0	0.103419
RootSys_A_LN	17.535833	19.562947	66.301924	7.349425	4.28294
Root_B_LN	20.469892	30.456003	88.313588	11.119311	6.89142
Leaf_B_LN	0.395379	1.008726	0	0	0.163498
seeds_5dai_mut	0.8054	2.283206	0.864774	0.08219	0.209253
LowerLeaf_C_LN	0.11829	0.663827	0.030114	0.040951	0.584493
UpperLeaf_C_LN	0.169448	0.689793	0.034054	0.091273	1.644225
Seeds_12dap	0.702918	0.017641	0	0	0
Seeds_12dap_mut	0.435709	0.018303	0	0	0.019466
Root_F_LN	26.992186	40.734148	84.426912	11.633774	12.305869
Nodule_G_LN	1.198856	4.213681	14.114144	2.676233	0.475798
Nodule_A_LN	0.952528	3.415073	10.971314	1.997266	0.56073
Nodule_B_LN	2.268257	4.899993	15.801208	1.583306	0.741521
ApicNode_B_LN	0.185706	0.215687	0	0	0.01761
Flower_B_LN	0.522123	0.324187	0.04075	0	0
Pods_C_LN	0.225617	0.065138	0	0	0.006904

TPM

low high

Figure S7. The expression level of PsSRF and candidate genes of PsFOS and PsFAT in pea plants using data from Alves-Carvalho et al. (2015). According to the previous study, stage A represents 7–8 nodes, 5–6 opened leaves; stage B represents the start of flowering; stage C represents 20 days after the start of flowering; stage D represents germination, 5 days after imbibition; stage E represents 12 days after pollination; stage F represents 8 days after sowing; and stage G represents 18 days after sowing, i.e., 10 days after inoculation. LN indicates low nitrate, and HN indicates high nitrate. The correlation efficiency of each candidate gene to PsSRF is shown by the PCC value.

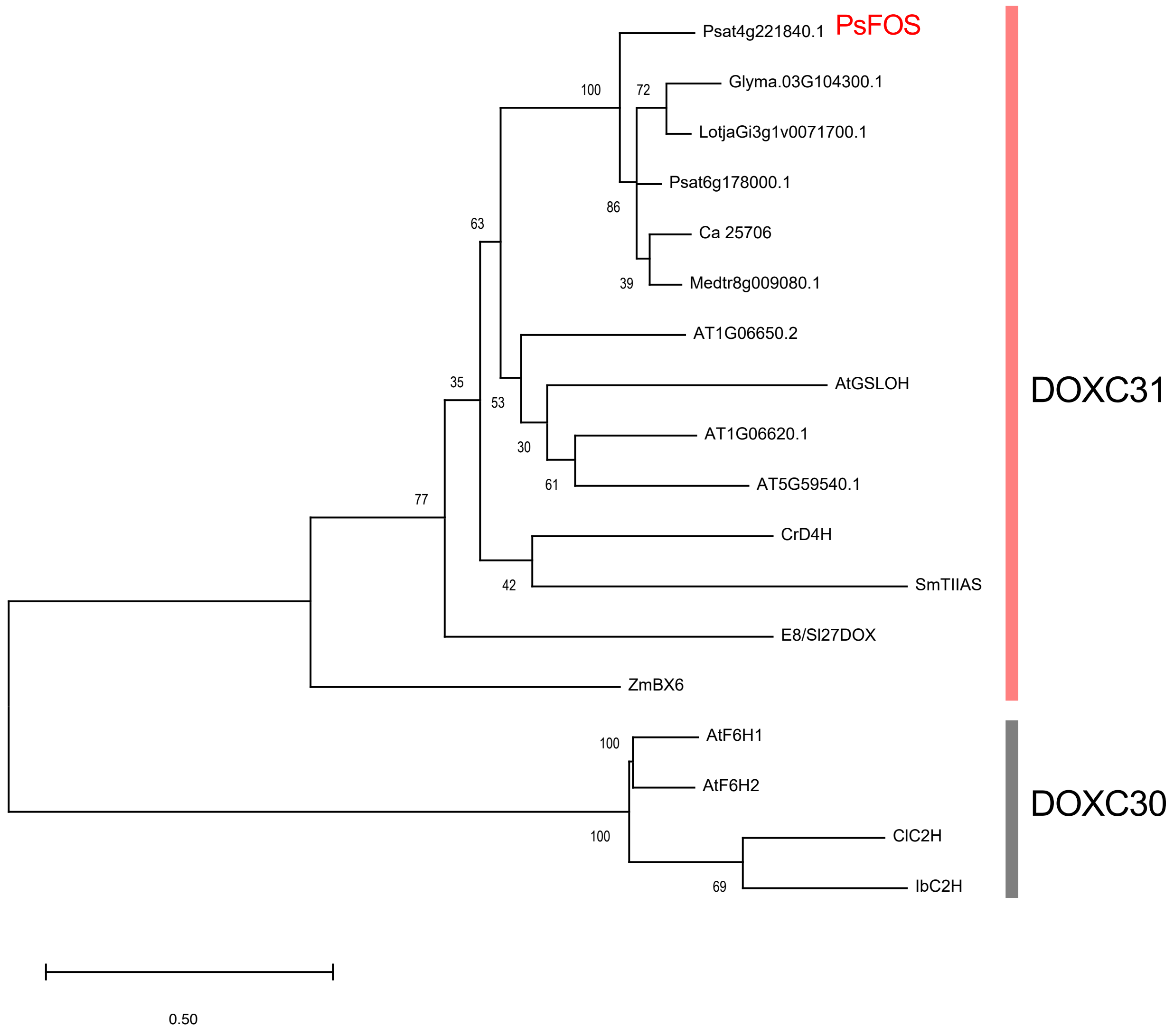

Figure S8. Phylogenetic analysis of PsFOS. Using the amino acid sequences of DOX31 enzymes reported previously and the homologs of PsFOS showing amino acid identity greater than 65%, the phylogenic tree was constructed by the maximum-likelihood method. DOXC30 enzymes were used as outgroups. Bootstrap values based on 1,000 replicates are shown at the branching points. The scale bar indicates 0.5 substitutions per position in the sequence.

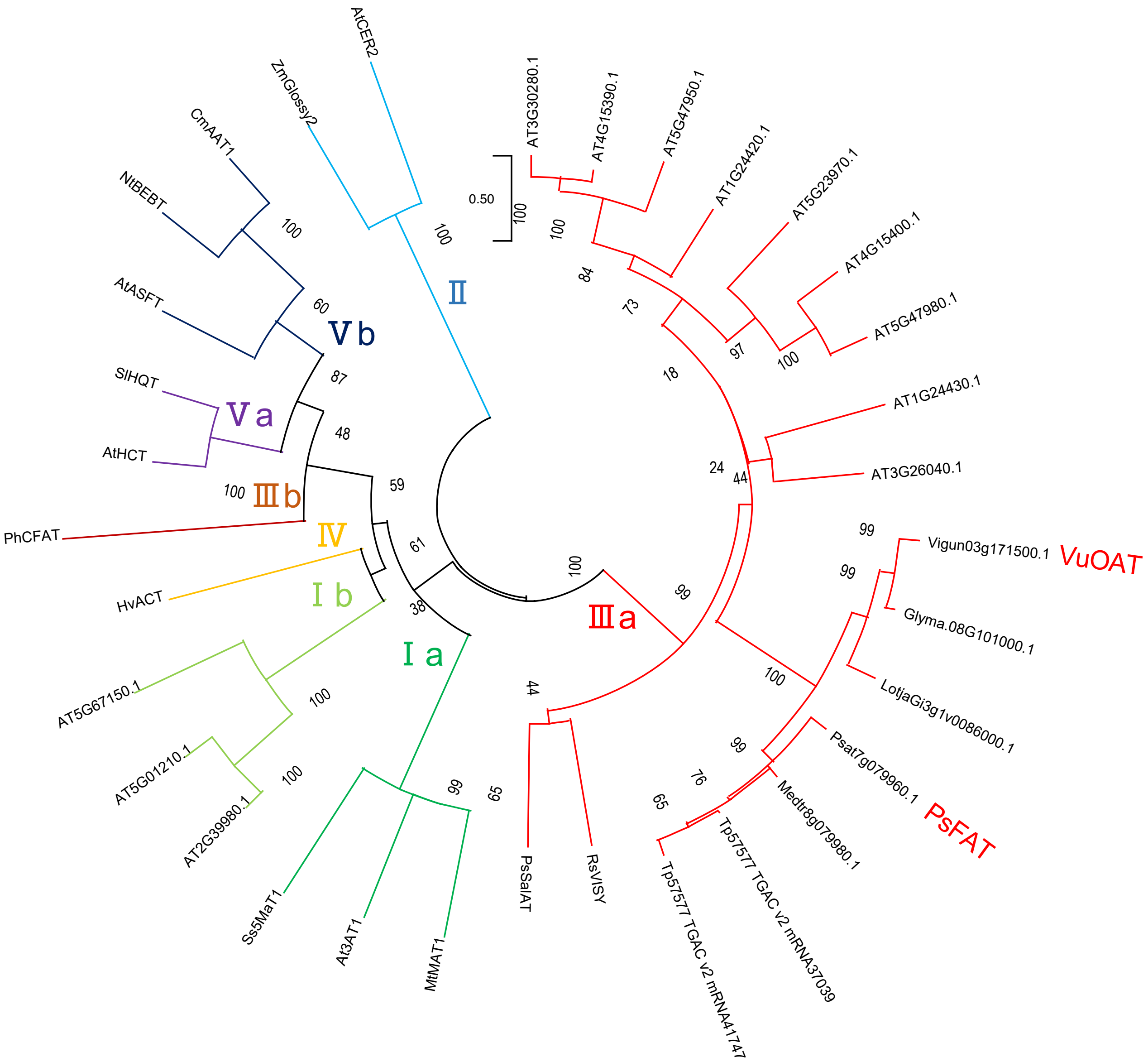

Figure S9. Phylogenetic analysis of PsFAT and VuOAT. The analysis utilized the amino acid sequences of BAHD acyltransferases that belong to the Ia, Ib, II, IIIa, IIIb, IV, Va, and Vb clades (Tuominen et al., 2011). Additionally, BAHD acyltransferases that showed an amino acid identity greater than 60% to PsFAT or VuOAT were included. The phylogenetic tree was constructed using the maximum-likelihood method. Bootstrap values, based on 1,000 replicates, are displayed at the branching points. The scale bar represents 0.5 substitutions per position in the sequence.

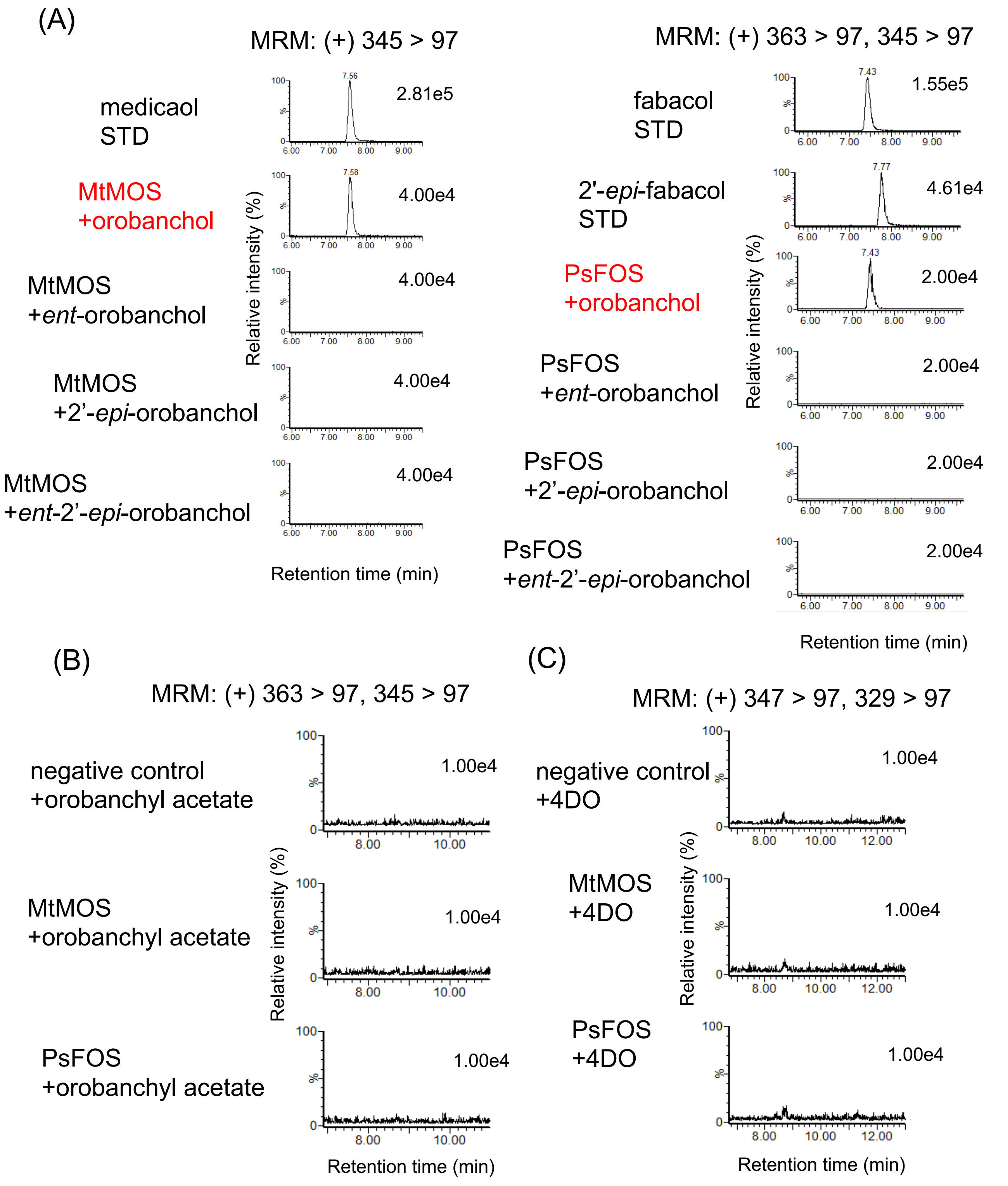

Figure S10. The substrate specificity of MtMOS and PsFOS. (A) Enzyme activity for orobanchol stereoisomers of MtMOS and PsFOS. (B) Enzyme activities toward orobanchyl acetate of MtMOS and PsFOS. The enzyme product, whose retention time is between that of orobanchyl acetate and fabacol, was monitored by MRM transitions at 345 > 97 and 363 > 97. (C) Enzyme activities toward 4DO of MtMOS and PsFOS. The enzyme product whose retention time was between that of 4DO and fabacol was monitored by MRM transitions at 329 > 97 and 347 > 97. Buffer without purified enzymes was used as a negative control.

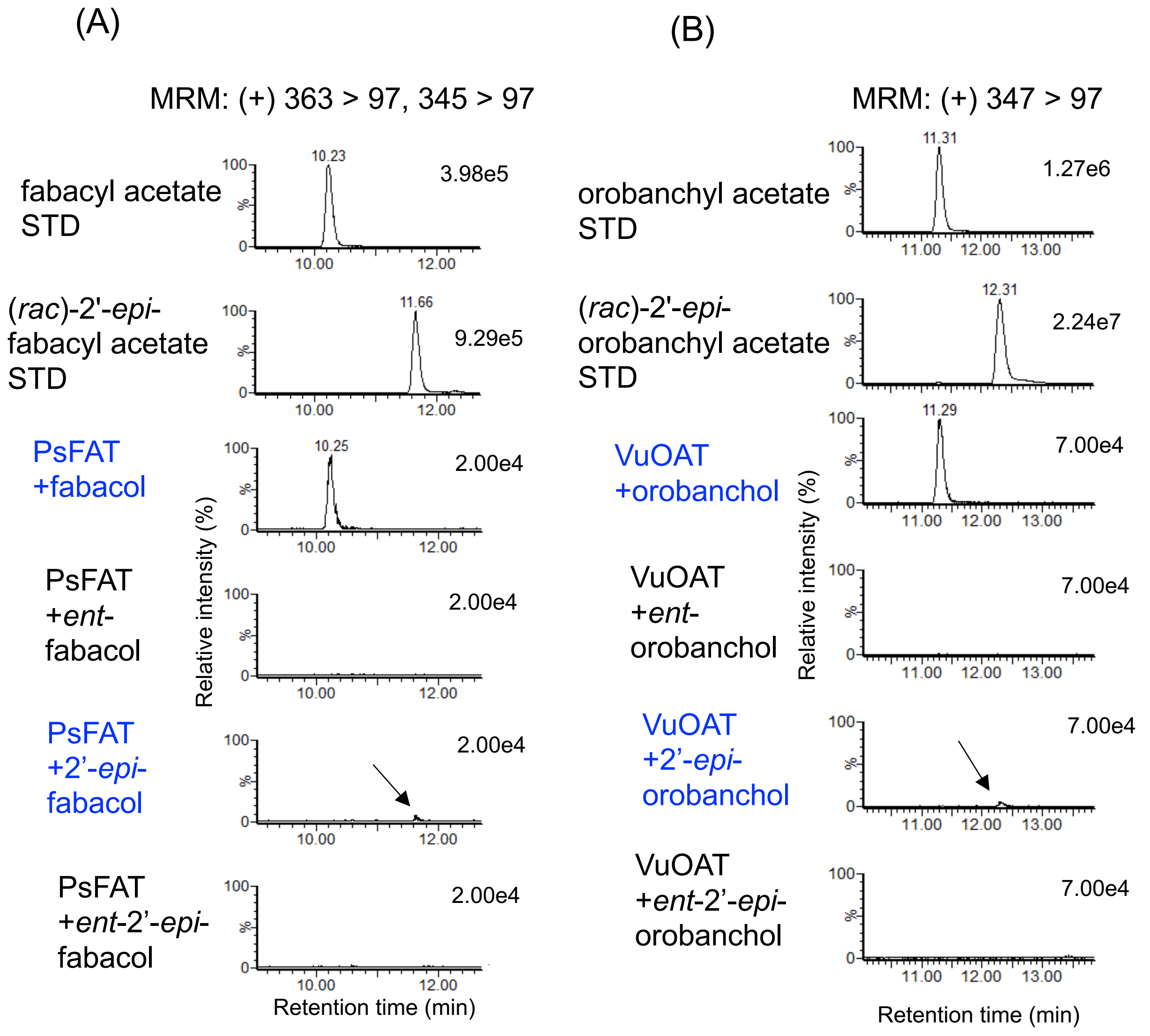

Figure S11. The substrate specificity of PsFAT and VuOAT. (A) Enzyme activity of PsFAT toward fabacol stereoisomers. The weak peak of the enzyme product, 2‘-epi-fabacyl acetate, is indicated by an arrow symbol. (B) Enzyme activity of VuOAT toward orobanchol stereoisomers. The weak peak of the enzyme product, 2‘-epi-orobanchyl acetate, is indicated by an arrow.

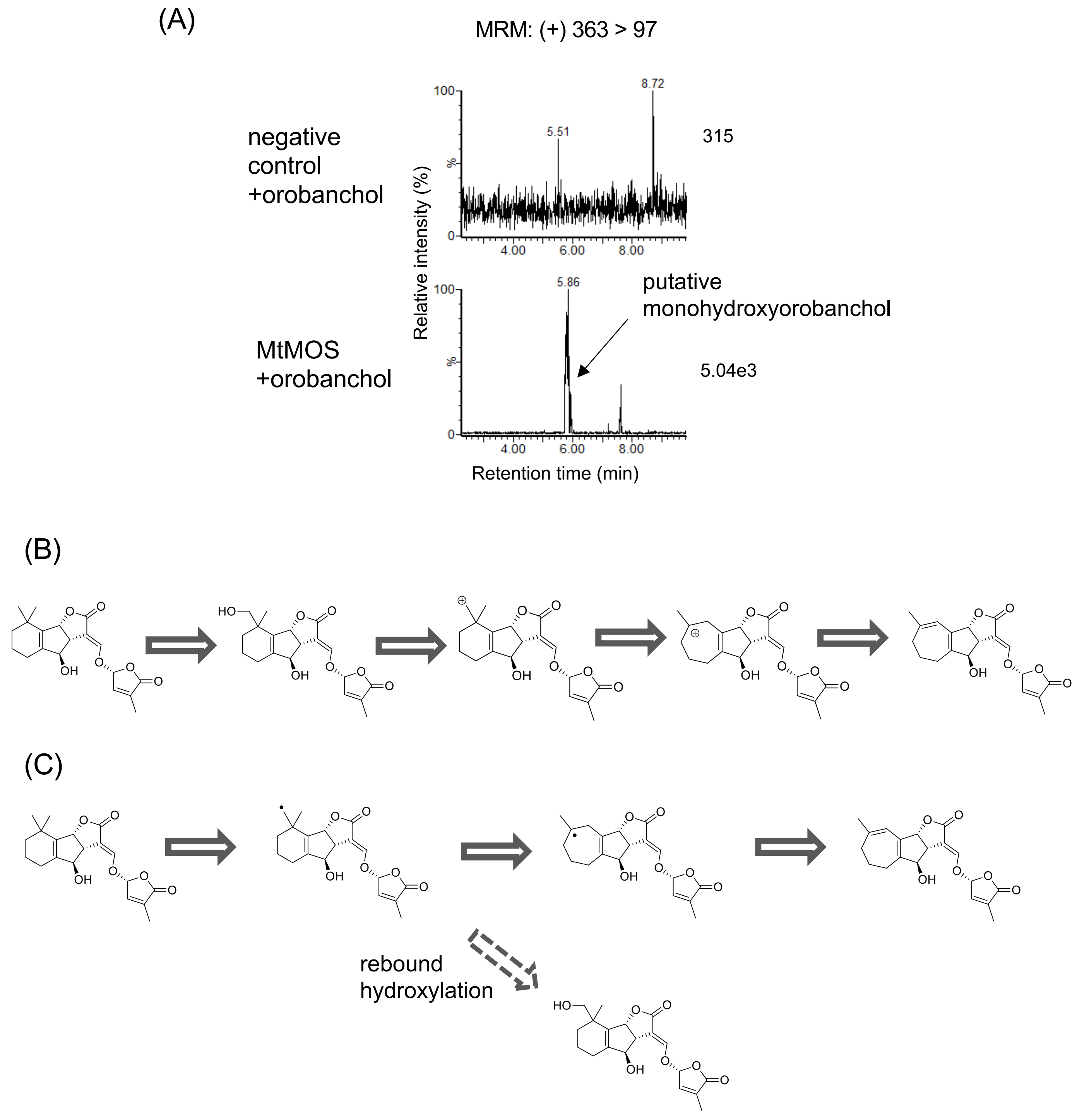

Figure S12. Putative reaction mechanism of MtMOS. (A) The detection of putative monohydroxyorobanchol in the enzyme reaction of MtrMOS. (B) The putative ring expansion mechanism of MtMOS is initialized by the hydroxylation of orobanchol, followed by the elimination of the hydroxy group. (C) Putative radical-based ring expansion mechanism.
